# Enhancement of ergothioneine production in *Corynebacterium glutamicum* by increasing osmotic pressure

**DOI:** 10.1007/s00253-025-13639-3

**Published:** 2025-12-08

**Authors:** Yuno Takahashi, Takashi Hirasawa

**Affiliations:** 1 School of Life Science and Technology, Institute of Science Tokyo, 4259 Nagatsuta-cho, Midori-Ku, Yokohama, Kanagawa 226-8501 Japan; 2 Present Address: Department of Natural Environmental Studies, Graduate School of Frontier Sciences, The University of Tokyo, 5-1-5 Kashiwanoha, Kashiwa, Chiba, 277-8564 Japan

**Keywords:** Ergothioneine, *Corynebacterium glutamicum*, Osmotic pressure, Betaine

## Abstract

**Abstract:**

Ergothioneine (EGT), which exhibits strong antioxidant properties, is an amino acid derivative with a betaine structure. Currently, studies have examined EGT import systems and its physiological roles in various organisms. Despite the broad applicability of EGT, industrial production with high productivity has not yet been achieved. In this study, we aimed to develop fermentative production methods for EGT using *Corynebacterium glutamicum* as a host and successfully achieved the highest yield of EGT (459 mg L^−1^) reported to date. A cysteine-producing strain *C. glutamicum* CYS-2, which was constructed in a previous study, was engineered to enhance the biosynthesis of histidine and *S*-adenosylmethionine, both of which, along with cysteine, are required for EGT production. Additionally, heterologous metabolic pathways for EGT biosynthesis from *Mycolicibacterium smegmatis* and *Methylobacterium pseudosasicola* were introduced into the engineered strain, which was designated CHS2. In batch cultivation, the CHS2 strain produced more EGT than the CYS-2 strain harboring the same EGT biosynthesis pathway. Interestingly, batch cultivation of the CHS2 strain under high osmotic pressure conditions prolonged the time for EGT production and increased the intracellular accumulation of EGT. These results suggest that increasing osmotic pressure together with engineering the biosynthesis of cysteine, histidine, and *S*-adenosylmethionine is an effective strategy for enhancing EGT production in recombinant *C. glutamicum* harboring heterologous EGT biosynthesis pathways.

**Key points:**

• *Ergothioneine production in C. glutamicum was enhanced by metabolic engineering*.

• *Osmotic pressure affects ergothioneine production in engineered C. glutamicum*.

• *Ergothioneine may function as a compatible solute in C. glutamicum*.

**Supplementary Information:**

The online version contains supplementary material available at 10.1007/s00253-025-13639-3.

## Introduction

Ergothioneine (EGT) is a sulfur-containing amino acid widely recognized for its antioxidant properties. This compound effectively scavenges reactive oxygen species in organisms and protects cells from oxidative damage. In humans, EGT accumulates in certain organs, such as the liver and kidneys, through dietary intake via the EGT-specific transporter OCTN1 (Gründemann et al. 2005). Recent research suggests that EGT contributes to lifespan extension through multiple mechanisms in *Mus musculus* and *Caenorhabditis elegans* (Katsube et al. 2024). Interestingly, *Streptococcus pneumoniae*, a major cause of pneumonia (Zhang et al. 2022), and *Helicobacter pylori* associated with stomach cancer (Dumitrescu et al. 2022) also transport EGT specifically into their cells through ATP-binding cassette transporters, and EGT is suggested to protect these pathogens against the host immune system. Therefore, understanding the physiological roles of EGT is crucial, not only in humans but also in diverse organisms.

Despite potential applications of EGT, its industrial production remains insufficient to meet increasing demand. As its range of applications expands, current methods for mass production, such as extraction from mushrooms, remain time-consuming and yield low harvests, making EGT an expensive compound. Recently, fermentative production of EGT has been proposed as an efficient and eco-friendly alternative. Various microorganisms have been explored for EGT production as host species including *Escherichia coli* (Osawa et al. 2018; Tanaka et al. 2019; Kamide et al. 2020; Zhang et al. 2024), *Corynebacterium glutamicum* (Kim et al. 2022; Hirasawa et al. 2023), *Bacillus licheniformis* (Liu et al. 2025), *Methylobacterium* sp. 22 A (Alamgir et al. 2015), *Mycolicibacterium neoaurum* (Liu et al. 2022; Ding et al. 2024), *Saccharomyces cerevisiae* (van der Hoek et al. 2019, 2022b; Yu et al. 2020), *Yarrowia lipolytica* (van der Hoek et al. 2022a), *Rhodotorula toruloides* (Liu et al. 2024), and *Aspergillus oryzae* (Takusagawa et al. 2019).

**Fig. 1:**
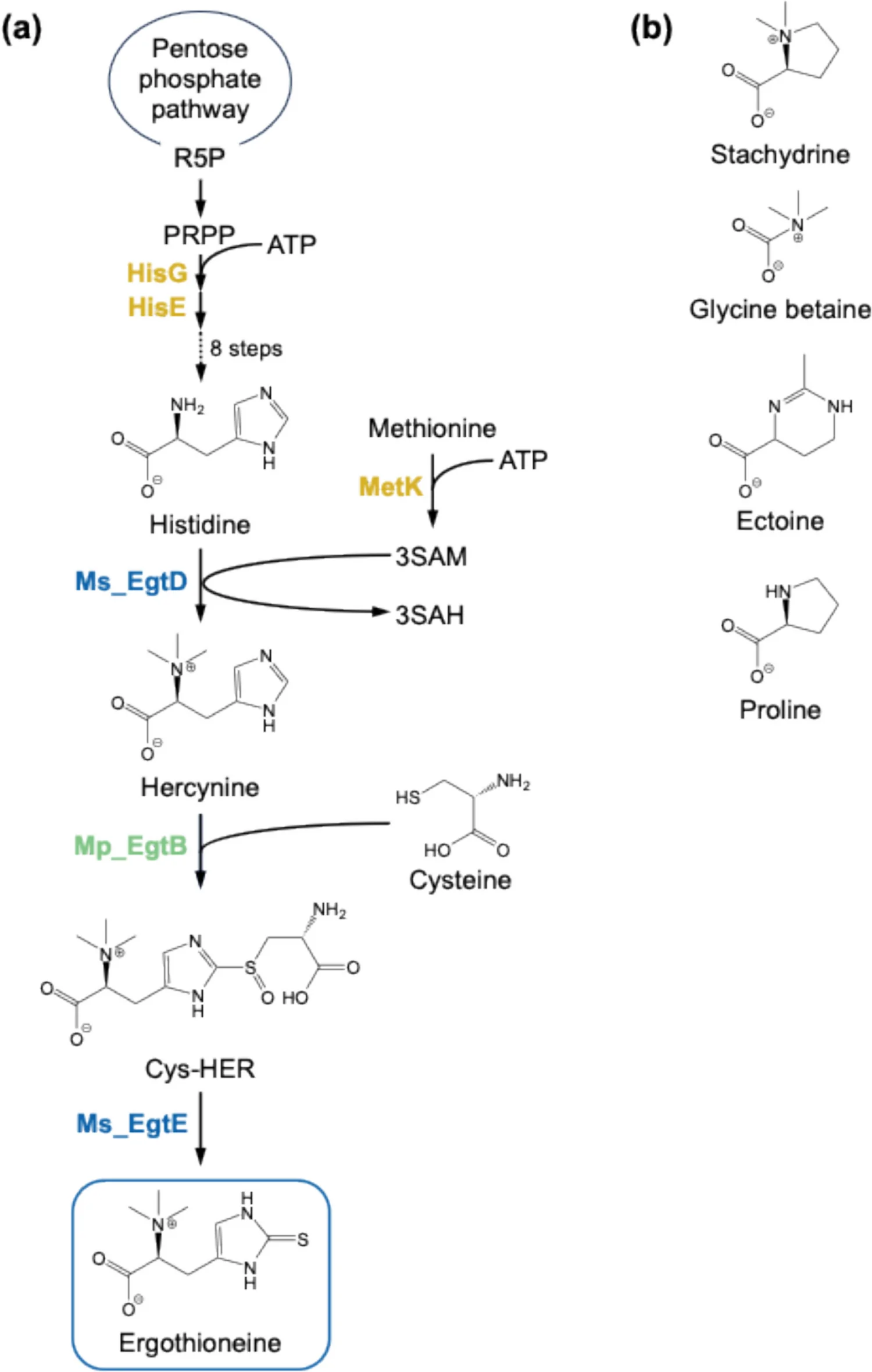
Metabolic pathways for EGT production implemented into *C. glutamicum* in the present study (**a**) and chemical structure of representative betaines (**b**). R5P, ribose-5-phosphate; PRPP, phosphoribosyl pyrophosphate; SAM, *S*-adenosylmethionine; SAH, *S*-adenosylhomocysteine; Cys-HER, Hercynylcysteine sulfoxide

For the fermentative production of EGT by microorganisms that do not naturally biosynthesize EGT, the necessary biosynthesis genes have been introduced into the host species by genetic engineering. EGT biosynthesis genes have been identified in several species, including *Mycobacterium* (Seebeck 2010), cyanobacteria (Liao and Seebeck 2017), fission yeast (Pluskal et al. 2014), fungi (Bello et al. 2012), and *Basidiomycetes* (Yang et al. 2020). Two distinct EGT biosynthetic pathways have been chosen for introducing EGT biosynthesis genes into host species. In these pathways, reactions for hercynylcysteine sulfoxide synthesis differ. In one pathway, hercynine is converted to hercynyl-γ-glutamylcysteine sulfoxide using γ-glutamylcysteine as a sulfur donor and then further converted to hercynylcysteine sulfoxide. In another pathway, hercynine is directly converted to hercynylcysteine sulfoxide using cysteine as a sulfur donor. Osawa et al. (2018) reported that introducing the EGT biosynthesis pathway from *Mycolicibacterium smegmatis* (formerly *Mycobacterium smegmatis*), which consists of five enzymes (EgtA, EgtB, EgtC, EgtD, and EgtE) and uses γ-glutamylcysteine as a sulfur donor to yield hercynylcysteine sulfoxide by EgtB, into *E. coli* resulted in fermentative EGT production and identified the EgtB-catalyzed reaction as a bottleneck in EGT biosynthesis. Kamide et al. (2020) demonstrated that EgtBs from *Methylobacterium brachiatum* and *Methylobacterium pseudosasicola* catalyze the direct conversion of hercynine to hercynylcysteine sulfoxide using cysteine as a sulfur donor. The introduction of EgtB from *M. brachiatum* or *M. pseudosasicola* into the EGT-producing *E. coli* strains effectively bypassed this bottleneck, leading to 657 mg L^−1^ of EGT in 192 h in batch cultivation. Hirasawa et al. (2023) reported EGT production by *C. glutamicum*, which was originally isolated as a glutamic acid producer (Kinoshita et al. 1957) and has been used as a host strain for the production of various amino acids, organic acids, and proteins. In this report, the *C. glutamicum* recombinant strain harboring the EGT biosynthesis pathway, which consists of EgtB from *Methylobacterium* and EgtD and EgtE from *M. smegmatis* (Fig. 1a), secreted approximately 100 mg L^−1^ of EGT after 336 h in batch cultivation.

The EGT-specific transporters identified in *S. pneumoniae* (Zhang et al. 2022) and *H. pylori* (Dumitrescu et al. 2022) were initially categorized as quaternary amine transporters responsive to environmental osmolarity. In most bacteria, betaines accumulate in cells as compatible solutes, protecting against osmotic stress by reducing turgor pressure or stabilizing proteins under ionic strength (Arakawa and Timasheff 1985). In the study of OCTN1 identified as a human EGT importer (Gründemann et al. 2005), stachydrine (proline betaine) was used as the lead substrate due to its structural similarity to EGT (Fig. 1b). This study reported that OCTN1 has weak but detectable betaine transport activity. Based on these observations, we hypothesized that betaine transporters in a host strain expressing EGT biosynthetic genes may affect EGT production. It has been reported that *C. glutamicum* has four osmoresponsive transporters: BetP (Peter et al. 1996), LcoP (Steger et al. 2004), EctP (Peter et al. 1998), and ProP (Peter et al. 1998). These transporters allow *C. glutamicum* to accumulate betaine under high osmotic pressure conditions.

In the present study, we metabolically engineered EGT-producing strains of *C. glutamicum* which were constructed in our previous study (Hirasawa et al. 2023) to enhance biosynthesis of the substrates for EGT production, histidine and *S*-adenosylmethionine (SAM), for improving EGT productivity. Moreover, based on the potential physiological role of EGT in *C. glutamicum* and its chemical structure, we investigated the impact of osmotic pressure on EGT production by measuring intracellular and extracellular EGT levels, exploring previously unexamined aspects of EGT production.

## Materials and methods

### Bacterial strains, plasmids, and media

*C. glutamicum* wild-type NBRC 12168 (synonymous with the type strain ATCC 13032) and the cysteine-producing strain CYS-2 were used as host species for EGT production. The CYS-2 strain was constructed in our previous study by introducing the mutant *cysE* gene (T94A) under the *tuf* promoter into a neutral site of the *C. glutamicum* genome and deleting the 3′ region of the *serA* gene, along with disruption of *cysE* and *aecD* genes (Kishino et al. 2019). For the secreted production of ergothionase (EGTase) from *Burkholderia* sp. HME13 used for EGT quantification, the *C. glutamicum* wild-type strain ATCC 13869 expressing EGTase with the CspB signal sequence from *C. glutamicum* ATCC 13869 (Hirasawa et al. 2023) was used. *E. coli* JM109 [*recA*1 *endA*1 *gyrA*96 *thi hsdR*17(r_K_^–^ m_K_^+^) e14^–^(*mcrA*^–^) *supE*44 *relA*1 Δ(*lac-proAB*)/F´(*traD*36 *proAB*^+^ *lacI*^q^ *lacZ*ΔM15)] was used for plasmid construction.

The plasmid pK18*mobsacB* (Schäfer et al. 1994) was used to construct plasmids for introducing the *tuf* promoter upstream of the *hisG* and *metK* genes, replacing the base in the *hisG* gene, and disrupting genes encoding betaine transporters in *C. glutamicum*. The pECt-Mp_egtB-Ms_egtDE carrying EGT biosynthesis genes *egtB* from* M. pseudosasicola* (Mp_*egtB*) and *egtDE* from *M. smegmatis* (Ms_*egtDE*) under the *C. glutamicum tuf* promoter (Hirasawa et al. 2023) was used to construct EGT production strains of *C. glutamicum*. The pECt-Mp_egtB-Ms_egtDE-transformed NBRC 12168 and CYS-2 strains were designated N and C, respectively.

Both *E. coli* and *C. glutamicum* were cultured in Lennox (L) medium [10 g L^−1^ hipolypepton (Shiotani M. S. Co., Ltd., Hyogo, Japan), 5 g L^−1^ dried yeast extract D-3H (Shiotani M. S. Co., Ltd.), 5 g L^−1^ NaCl, 1 g L^−1^ glucose; pH 7.0] or brain heart infusion sorbitol medium [37 g L^−1^ brain heart infusion (Becton, Dickinson and Company, Franklin Lakes, NJ), 91 g L^−1^ sorbitol] during the construction of recombinant strains. For EGT production experiments, L medium was used for preculture, and then a semisynthetic medium [80 g L^−1^ glucose, 30 g L^−1^ (NH_4_)_2_SO_4_, 3 g L^−1^ Na_2_HPO_4_·12H_2_O, 6 g L^−1^ KH_2_PO_4_, 2 g L^−1^ NaCl, 84 mg L^−1^ CaCl_2_, 3.9 mg L^−1^ FeCl_3_, 0.9 mg L^−1^ ZnSO_4_·7H_2_O, 0.3 mg L^−1^ CuCl_2_·2H_2_O, 5.56 mg L^−1^ MnSO_4_·5H_2_O, 0.1 mg L^−1^ (NH_4_)6Mo_7_O_24_·4H_2_O, 0.3 mg L^−1^ Na_2_B_4_O_7_·10H_2_O, 0.4 g L^−1^ 16MgSO_4_·7H_2_O, 40 mg L^−1^ FeSO_4_·7H_2_O, 0.5 mg L^−1^ thiamin hydrochloride, 0.1 g L^−1^ ethylenediamine-*N*, *N*, *N*´, *N*´-tetraacetic acid disodium salt dihydrate, 0.03 mg L^−1^ D-biotin, 10 g L^−1^ dried yeast extract D-3H, 25 g L^−1^ CaCO_3_; pH 7.2] was used for main culture. For secreted production of EGTase, CM2G medium [10 g L^−1^ hipolypepton, 10 g L^−1^ dried yeast extract D-3H, 5 g L^−1^ NaCl, 5 g L^−1^ glucose, 0.2 g L^−1^ methionine; pH 7.2] was used for preculture, and then MMTG medium [120 g L^−1^ glucose, 2 mg L^−1^ dried yeast extract, 1.5 g L^−1^ KH_2_PO_4_, 30 g L^−1^ (NH_4_)_2_SO_4_, 3 g L^−1^ MgSO_4_·7H_2_O, 0.03 g L^−1^ MnSO_4_·5H_2_O, 0.03 g L^−1^ FeSO_4_·7H_2_O, 0.45 mg L^−1^ thiamin hydrochloride, 0.45 mg L^−1^ D-biotin, 50 g L^−1^ CaCO_3_; pH 7.0] was used for the main culture. As needed, 10 and 20 mg L^−1^ kanamycin for *C. glutamicum* and *E. coli*, respectively, and 200 g L^−1^ sucrose were added to the media.

### Engineering of biosynthesis of histidine and SAM in *C. glutamicum*

According to Zhang et al. (2012), the introduction of a mutation that replaces the 270th alanine residue with aspartic acid (designated A270D) in ATP phosphoribosyl transferase encoded by *hisG* desensitizes this enzyme to feedback inhibition by histidine. To engineer histidine biosynthesis in *C. glutamicum*, this mutation was introduced into the *hisG* locus in the *C. glutamicum* genome. Firstly, 500-bp upstream and downstream of the mutation site in the *hisG* open reading frame (ORF) were amplified by polymerase chain reaction (PCR) using KOD One PCR Master Mix (Toyobo Co., Ltd., Osaka, Japan). The GAT codon for aspartic acid instead of GCC for alanine was included in the designed primers (Supplementary Table S1) and then connected by overlap extension PCR, creating an approximately 1000-bp DNA fragment with the mutation. The connected fragment was then cloned into the EcoRI-BamHI sites of pK18*mobsacB*, generating the pK18*mobsacB*_hisGA270D plasmid. After confirming the sequence of the cloned fragment, the plasmid was introduced into the *C. glutamicum* wild-type strain NBRC 12168 and its cysteine-producing strain CYS-2. To select cells in which the plasmid was integrated into the genome by homologous recombination, the transformants were spread on an L plate containing kanamycin and cultivated at 30 °C for 3 d. A single colony of the transformant was inoculated into 5 mL of L medium containing kanamycin and incubated at 30 °C overnight. A small aliquot of this culture was inoculated into L medium without kanamycin addition and cultured at 30 °C overnight. Cells that lost pK18*mobsacB* from the genome via a 2nd homologous recombination event should become kanamycin-sensitive and sucrose-resistant. Therefore, the culture was diluted and spread on an L plate containing 20% sucrose to select the sucrose-resistant strains. The recombinant strains were verified to have lost kanamycin resistance by the replica plating method. Finally, a strain carrying the A270D mutation in the *hisG* locus was obtained by confirming the introduction of the desired mutation with DNA sequencing analysis. The pECt-Mp_egtB-Ms_egtDE-transformed *hisG* mutant strains constructed from NBRC 12168 and CYS-2 were designated H2 and CH2, respectively.

To overexpress the *hisEG* operon in *C. glutamicum*, the *tuf* promoter sequence was inserted just upstream of the *hisEG* operon. The 200-bp DNA fragment of the *tuf* promoter and 500-bp DNA regions upstream and downstream of the initiation codon of the *hisE* gene were amplified from the genome of *C. glutamicum* using the primer sets listed in Supplementary Table S1. The three amplified fragments were connected to generate one DNA fragment using overlap extension PCR in the following order: upstream fragment, *tuf* promoter, and downstream fragment. The final 1200-bp DNA fragment was inserted into the EcoRI-BamHI sites of pK18*mobsacB*. The resulting plasmid which was designated pK18*mobsacB*_P_*tuf*__*hisEG* was introduced into the *C. glutamicum* NBRC 12168 and CYS-2 strains carrying the A270D mutation in the *hisG* locus. Target recombinant strains were obtained by inducing double homologous recombination events as described above and insertion of the *tuf* promoter sequence just upstream of the *hisEG* operon was confirmed by PCR. The pECt-Mp_egtB-Ms_egtDE-transformed *hisEG* overexpression strains constructed from H2 and CH2 were named H1 and CH1, respectively.

To engineer SAM biosynthesis in *C. glutamicum* according to Han et al. (2016), the *metK* gene encoding SAM synthetase was overexpressed by inserting the *tuf* promoter upstream of the *metK* ORF. An approximately 1000-bp region upstream and downstream of the initiation codon of *metK* was amplified from *C. glutamicum* genomic DNA using the primer sets listed in Supplementary Table S1. The amplified fragment was cloned into the EcoRI-BamHI sites of pK18*mobsacB*. Subsequently, inverse PCR was performed using the resulting plasmid pK18*mobsacB*_*metK* as a template. The primer sets contain the sequences homologous to the *tuf* promoter and can anneal to just upstream and downstream of the initiation codon of *metK*. The amplified fragment containing the pK18*mobsacB* sequence was connected to the PCR-amplified *tuf* promoter fragment using the seamless ligation cloning extract method (Motohashi 2015; Okegawa and Motohashi 2015b, 2015a). The resulting plasmid was named pK18*mobsacB*_P_*tuf*__*metK*. To construct the *metK*-overexpressing strain, pK18*mobsacB*_P_*tuf*__*metK* was introduced into *C. glutamicum* and double homologous recombination events were induced using the procedure described above. The pECt-Mp_egtB-Ms_egtDE-transformed *metK* overexpression strains obtained from NBRC 12168, C, H1, CH1, H2, and CH2 were named S1, CS1, HS1, CHS1, HS2, and CHS2, respectively.

### Deletion of betaine transporter genes in *C. glutamicum*

The regions that were approximately 500-bp DNA fragments upstream and downstream of the ORFs of *betP*, *lcoP*, *ectP*, and *proP* genes encoding betaine transporters were amplified by PCR from the *C. glutamicum* genome. The primers for PCR are listed in Supplementary Table S2. Two fragments of each gene were connected and inserted into the appropriate restriction enzyme sites of pK18*mobsacB*, and the sequence of the cloned fragment was checked. The resulting plasmids, pK18*mobsacB*_betP_del, pK18*mobsacB*_lcoP_del, pK18*mobsacB*_ectP_del, and pK18*mobsacB*_proP_del were used to delete *betP*, *lcoP*, *ectP*, and *proP*, respectively. Each plasmid was introduced into the CYS-2 strain and kanamycin-resistant transformants were obtained. The deletion strains were obtained by inducing double homologous recombination events as described above, and the deletion of the target gene was verified by PCR. The pECt-Mp_egtB-Ms_egtDE-transformed resulting deletion strains were named CΔ*betP*, CΔ*lcoP*, CΔ*ectP*, and CΔ*proP*.

### EGT production in batch culture

For preparing the preculture, a single colony of EGT-producing strains of *C. glutamicum* was inoculated into 5 mL L medium and cultured at 30 ℃ for 1–2 days. Subsequently, 1.6 mL of the preculture was transferred into 40 mL semisynthetic medium in a 300-mL baffled flask for the main culture, and then, the culture was incubated at 30 ℃ with rotary shaking at 200 rpm. To create high osmotic pressure conditions, sorbitol or NaCl was added to the semisynthetic medium. During cultivation, optical density at 660 nm (OD_660_) was measured using a spectrophotometer UV-1280 (Shimadzu Corporation, Kyoto, Japan). When measuring OD_660_, samples were diluted with 0.2 N HCl to dissolve CaCO_3_.

Additionally, to analyze intracellular and extracellular EGT levels, 300 μL of the culture was collected in two microcentrifuge tubes and one of them was centrifuged to obtain culture supernatant and cell pellet. The culture supernatant was used to measure extracellular EGT and glucose concentrations. The cell pellet was resuspended in 300 μL of 10 mM sodium phosphate buffer (pH 7.2) and boiled for 20 min to extract EGT from the cells for measuring intracellular EGT concentration. The culture in another tube was boiled for 20 min. After boiling, the cell debris was removed by centrifugation and the EGT concentration in the supernatant, which corresponds to total EGT concentration (i.e., intracellular plus extracellular EGT concentration), was measured. Procedures for collecting samples are summarized in Supplementary Fig. S1.

### EGT production in fed-batch culture

To conduct fed-batch culturing, the CHS2 strain was precultured overnight, and the main culture was started in a 300-mL baffled flask containing 40 mL semisynthetic medium using the same procedures as those for batch culture. High osmotic pressure conditions in the main culture were created by adding sorbitol (0.5 M, final concentration) to the culture medium. After 48 h, 4 mL of 400 g L^−1^ glucose solution and 4 mL of 150 g L^−1^ ammonium sulfate solution were added to the culture. Both glucose and ammonium sulfate solutions were intermittently added every 24 h until the end of cultivation.

### Measurement of EGT and glucose concentration

The concentration of EGT was measured using an enzymatic method with EGTase as described by Hirasawa et al. (2023). The samples were diluted with 10 mM sodium phosphate buffer (pH 7.2). Next, 990 μL of the reaction mixtures containing the diluted samples or EGT standard in 10 mM sodium phosphate buffer (pH 7.2) was prepared, and the absorbance at 311 nm of each mixture (*A*_1_) was measured. Then, 10 μL of EGTase, which was prepared as described previously (Hirasawa et al. 2023), was added to each reaction mixture and the mixture was incubated at 30 ℃ for 1 h, followed by measurement of the absorbance at 311 nm (*A*_2_). From the calibration line, which was made using the *A*_2_ – *A*_1_ values for the EGT standard, the EGT concentration in the samples was determined using the *A*_2_ – *A*_1_ values for the culture samples.

The glucose concentration in the culture supernatants was measured using the Glucose CII test (Fujifilm Wako Pure Chemical Corporation, Osaka, Japan), following the instructions supplied by the manufacturer.

### Statistical analysis

EGT production data was statistically assessed by one-way analysis of variance (ANOVA) and Student’s *t*-test using software R (R Core Team 2025) and Microsoft Excel (Microsoft Corporation, Redmond, WA).

## Results

### Improvement of EGT secretion by engineering histidine and SAM biosynthesis

Considering the EGT biosynthesis pathways (Fig. 1a), enhancement of histidine and SAM supply to EGT biosynthesis was expected to improve EGT production. Trimethylation of histidine using SAM as a methyl donor to yield hercynine is the starting reaction of EGT biosynthesis (Fig. 1a). In addition, our previous studies revealed that the supplementation of histidine and SAM to the medium does not enhance EGT secretion in recombinant *C. glutamicum* (Hirasawa et al. 2023), and cysteine is toxic to *C. glutamicum* cells (Matsuhisa et al. 2025). Therefore, we focused on engineering the histidine and SAM biosynthesis in the wild-type strain NBRC 12168 and its cysteine production strain CYS-2, whose cysteine production levels reach more than 200 mg L^−1^ at 12 h (Supplementary Fig. S2), and evaluated the effect of this modulation on EGT secretion by the strains harboring EgtB from *M. pseudosasicola* and EgtD and EgtE from *M. smegmatis*. Preculture of the H1 and CH1 strains needed to be conducted for 48 h because their growth was slower than that of the other strains (data not shown). During the main culture, OD_660_ values of the EGT-producing recombinant strains of *C. glutamicum* peaked at 24 h and then decreased from 48 to 72 h (Supplementary Fig. S3). After 72 h, OD_660_ values remained almost constant until the end of the cultivation.

**Fig. 2:**
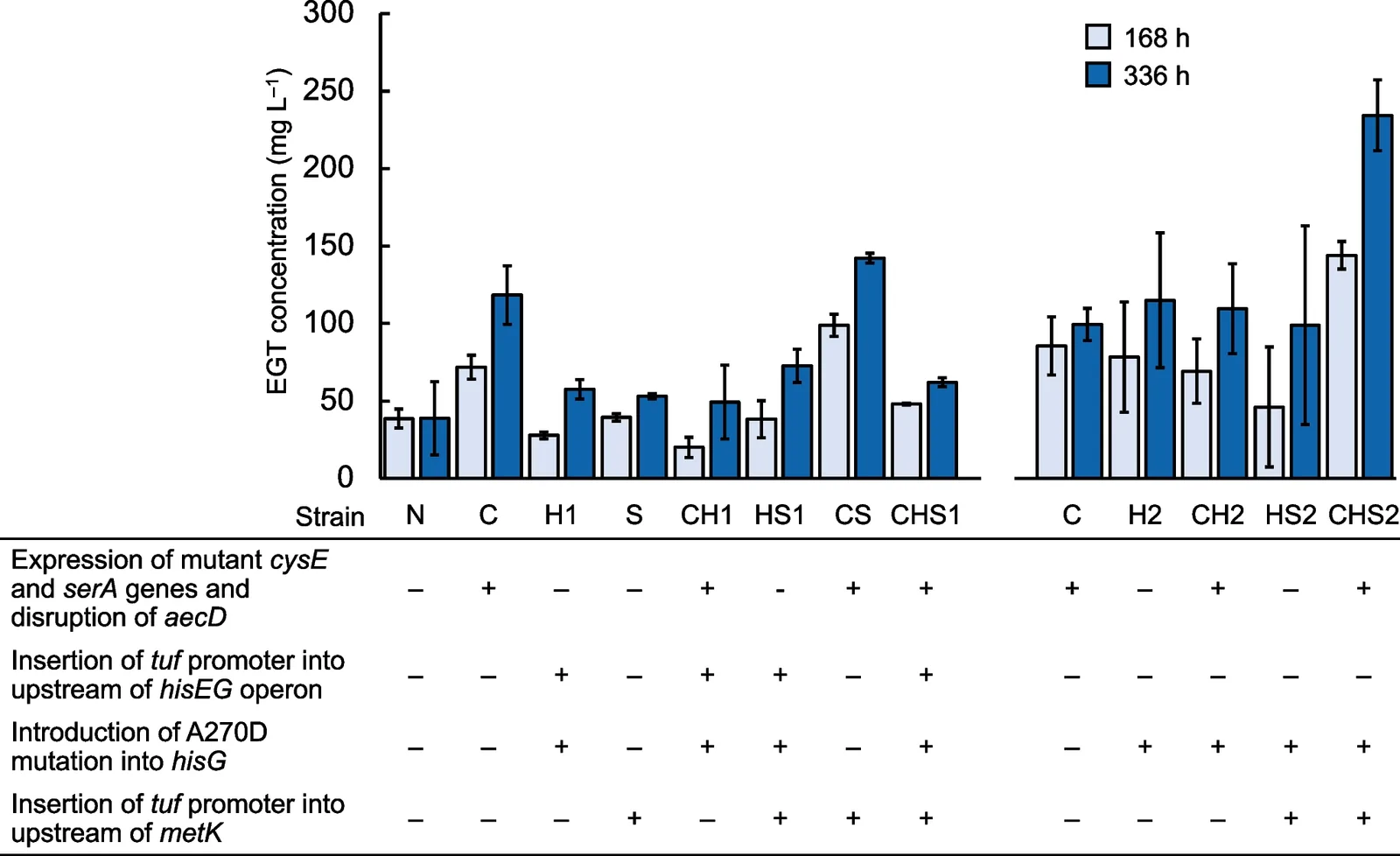
Effect of engineering of histidine and SAM biosynthesis on EGT secretion by *C. glutamicum*. + and − represent the engineering strategies applied and not applied, respectively, to the NBRC 12168 and C strains. After the engineering, the strains were transformed with the pECt-Mp_egtB-Ms_egtDE carrying EGT biosynthesis genes. The resulting strains were cultured for 336 h and EGT concentration in the culture supernatant obtained at 168 h (light blue bars) and 336 h (dark blue bars) was measured. The experiment was conducted in triplicate. Average ± standard deviation is shown

Figure 2 shows the EGT concentration in the supernatant of the main culture (i.e., EGT secretion levels) at 168 and 366 h (1 and 2 weeks, respectively). The data of EGT secretion by all the strains examined were assessed with one-way ANOVA (*p* = 2.7 × 10^−7^ and 3.3 × 10^−7^ for the data of EGT secretion at 168 and 336 h, respectively), revealing that the engineering of biosynthesis of cysteine, histidine and SAM significantly affected EGT secretion. In the N strain whose biosynthesis of cysteine, histidine and SAM was not engineered, 38.7 and 38.9 mg L^−1^ EGT was secreted at 168 and 366 h, respectively. In the C strain whose cysteine biosynthesis was engineered, 72 and 118 mg L^−1^ EGT was secreted at 168 and 366 h, respectively, as reported previously (Hirasawa et al. 2023). The EGT secretion by the CS1 strain, whose SAM biosynthesis was engineered by inserting *tuf* promoter just upstream of the ORF of *metK* encoding SAM synthetase together with the enhancement of cysteine biosynthesis, was higher than that by the C strain; the secretion levels were 99 and 142 mg L^−1^ at 168 and 336 h, respectively, but the difference in EGT secretion between C and CS1 strains was not significant (*p* = 7.5 × 10^−1^ and 1.0 for the data of EGT secretion at 168 and 336 h, respectively, in Tukey–Kramer post hoc test). In the host of the CS1 strain, SAM production was enhanced approximately 5 times at 48 h compared with that in the CYS-2 strain (i.e., host of the C strain) (Supplementary Fig. S4), confirming that the insertion of *tuf* promoter just upstream of *metK* ORF could enhance SAM production, and this would result in increased EGT secretion in the CS1 strain. Moreover, the EGT secretion by the CHS2 strain, whose biosynthesis of SAM and histidine was engineered by inserting *tuf* promoter just upstream of the *metK* ORF and introducing the mutation for A270D into the *hisG* gene encoding ATP phosphoribosyl transferase together with the enhancement of cysteine biosynthesis, was higher than that by the C strain; the secretion levels reached 144 and 234 mg L^−1^ at 168 and 366 h, respectively, which was approximately double that observed in the C strain. The difference in EGT secretion between C and CHS2 strains was significant (*p* = 1.6 × 10^−3^ and 1.3 × 10^−3^ for the data of EGT secretion at 168 and 336 h, respectively, in Tukey–Kramer post hoc test). In the host of the CHS2 strain, the production of both histidine and SAM was higher than that in the CYS-2 strain (Supplementary Fig. S4), suggesting that this phenomenon would result in increased EGT secretion in the CHS2 strain. These results indicate that engineering the biosynthesis of cysteine, histidine, and SAM is effective for enhancing EGT secretion.

Although a trend of increased EGT production in the CS1 strain, where *metK* was overexpressed and cysteine biosynthesis was enhanced, was observed, the introduction of the *hisG* mutation together with the insertion of the *tuf* promoter just upstream of the *hisEG* operon (i.e., the CH1 strain) did not improve EGT secretion. As shown in Supplementary Fig. S3, *metK* overexpression did not affect the growth of the strain, whereas the introduction of the *hisG* mutation with the insertion of the *tuf* promoter reduced cell growth, indicating that excess expression of the mutant *hisG* gene from the *tuf* promoter affects cell growth. However, the growth of HS1 and CHS1 cells was not inhibited. These results suggest that *metK* overexpression alleviates the growth reduction observed in the H1 and CH1 strains.

Considering that overexpression of the mutant *hisG* gene from the *tuf* promoter did not improve EGT secretion, the mutant *hisG* gene expression level needed to be optimized to improve cell growth. Therefore, we constructed the H2 strain in which the expression levels of the *hisEG* operon were expected to be lower than those of H1. The growth of the H2 strain was not reduced compared to that of the other strains (Supplementary Fig. S3). Moreover, EGT secretion by the CHS2 strain was the highest among the strains examined; EGT secretion reached 234 mg L^−1^ at 336 h. This result indicates that engineering histidine and SAM biosynthesis effectively improves EGT secretion in *C. glutamicum*.

### Effect of high osmotic pressure on EGT production by the C strain

*C. glutamicum* has osmoregulated uptake systems, betaine-carnitine-choline transporters (BCCTs), BetP, LcoP and EctP, and the proline importer ProP; these transporters import betaine or proline as compatible solutes in response to hyperosmotic stress (Morbach and Kramer 2003). It was hypothesized that BCCTs in *C. glutamicum* are activated under high osmotic pressure conditions, and as a result, EGT may accumulate in cells as a compatible solute because it is an amino acid with a betaine structure. To test this hypothesis, we cultured EGT-producing *C. glutamicum* under high osmotic pressure conditions and measured the intracellular and extracellular EGT concentrations. High osmotic pressure conditions were created by adding sorbitol, which is not assimilated as a carbon source by *C. glutamicum*, to the medium. In the present study, intracellular and extracellular concentrations of EGT per culture volume were determined.

**Fig. 3:**
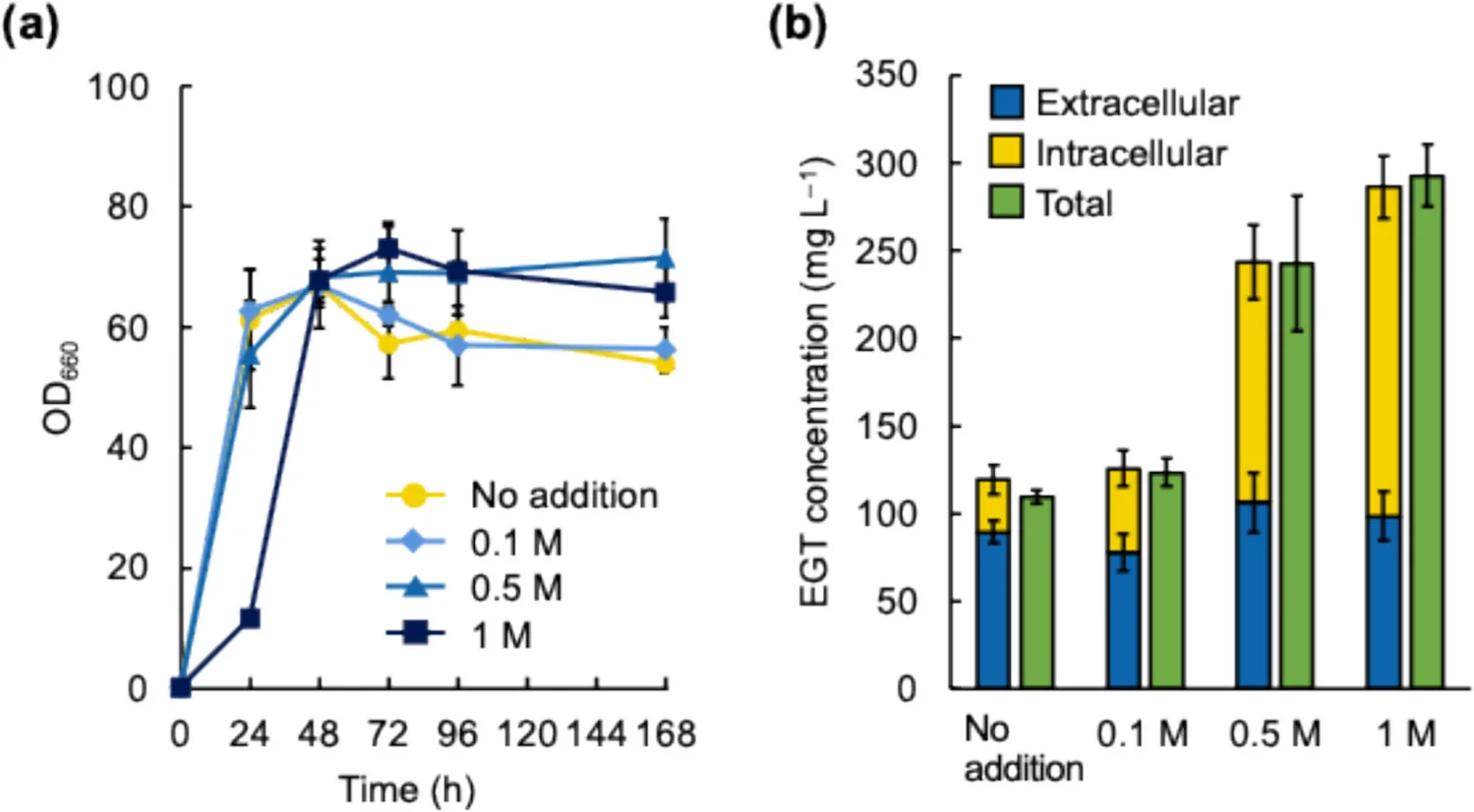
EGT production by the C strain under high osmotic pressure conditions. The strain was cultured under high osmotic pressure conditions and EGT production was measured. In **a**, cell growth of the strain without the addition of sorbitol (circles) and with the addition of 0.1 (diamonds), 0.5 (triangles), and 1 (squares) M sorbitol is shown. In **b**, extracellular EGT concentration (blue bars), intracellular EGT concentration (yellow bars) and total EGT concentration (green bars) at 168 h under different high osmotic pressure conditions created by sorbitol addition are shown. The experiment was conducted in triplicate. Average ± standard deviation is shown

First, the C strain was cultured in a semisynthetic medium containing 0.1, 0.5, or 1 M sorbitol for 168 h. The preculture was prepared overnight in the absence of sorbitol. Figure 3 shows the time course of cell growth and concentrations of intracellular and extracellular EGT per culture volume at 168 h, which correspond to EGT accumulation and secretion, respectively. Without sorbitol addition, the C strain reached the stationary phase at 24 h, whereas with 1 M sorbitol addition, it reached the stationary phase at approximately 48 h. No significant difference in the OD_660_ values in the stationary phase was observed with and without sorbitol addition.

Total EGT production, which is defined as intracellular plus extracellular EGT amounts per culture volume measured using boiled culture samples, and intracellular EGT production by the C strain were significantly affected by osmotic pressure, whereas extracellular EGT production was not; these phenomena were assessed by one-way ANOVA (*p* = 1.4 × 10^−5^, 4.3 × 10^−6^, and 1.1 × 10^−1^ for total, intracellular and extracellular EGT concentrations, respectively). Total EGT production was 110 mg L^−1^ without sorbitol addition. It increased to 242 and 293 mg L^−1^ with 0.5 or 1 M sorbitol addition, respectively. This increase in total EGT production by 0.5 and 1 M sorbitol was significant (*p* = 3.2 × 10^−4^ and 3.1 × 10^−5^, respectively, in Tukey–Kramer post hoc test). Although extracellular EGT concentrations were similar under all conditions tested, intracellular EGT accumulation levels varied substantially depending on the sorbitol concentration, reaching 30, 48, 137, and 187 mg L^−1^ without sorbitol addition and with 0.1, 0.5, and 1 M sorbitol addition, respectively. The increase in intracellular EGT concentration by 0.5 and 1 M sorbitol addition was significant (*p* = 1.3 × 10^−4^ and 7.2 × 10^−6^, respectively, in Tukey–Kramer post hoc test). These results indicate that osmotic pressure affects EGT production in *C. glutamicum* harboring the heterologous EGT biosynthesis pathway and that more EGT is produced and accumulated under high osmotic pressure conditions. However, EGT secretion by the C strain was not affected by increasing osmotic pressure.

To confirm whether our EGT quantification method using EGTase indeed detected only EGT in the samples from the C strain cultured under high osmotic pressure conditions, we cultured the CYS-2 strain carrying an empty vector pECt (Sato et al. 2008) with 0.5 M sorbitol addition and performed EGT quantification. As expected, EGT was not detected in the culture samples of the CYS-2/pECt strain (data not shown), suggesting that our EGT quantification method can detect EGT in the samples from the culture of the C strain.

Additionally, we observed the growth of the CYS-2/pECt and C strains under high osmotic pressure conditions created by NaCl addition as well as sorbitol addition. Here, 0.25 and 0.5 M NaCl were added to the semisynthetic medium because the osmotic pressures induced by 0.25 and 0.5 M NaCl are equivalent to those by 0.5 and 1 M sorbitol, respectively. As shown in Supplementary Fig. S5, the growth properties of both strains in the presence of NaCl were similar to those in the presence of sorbitol.

**Fig. 4:**
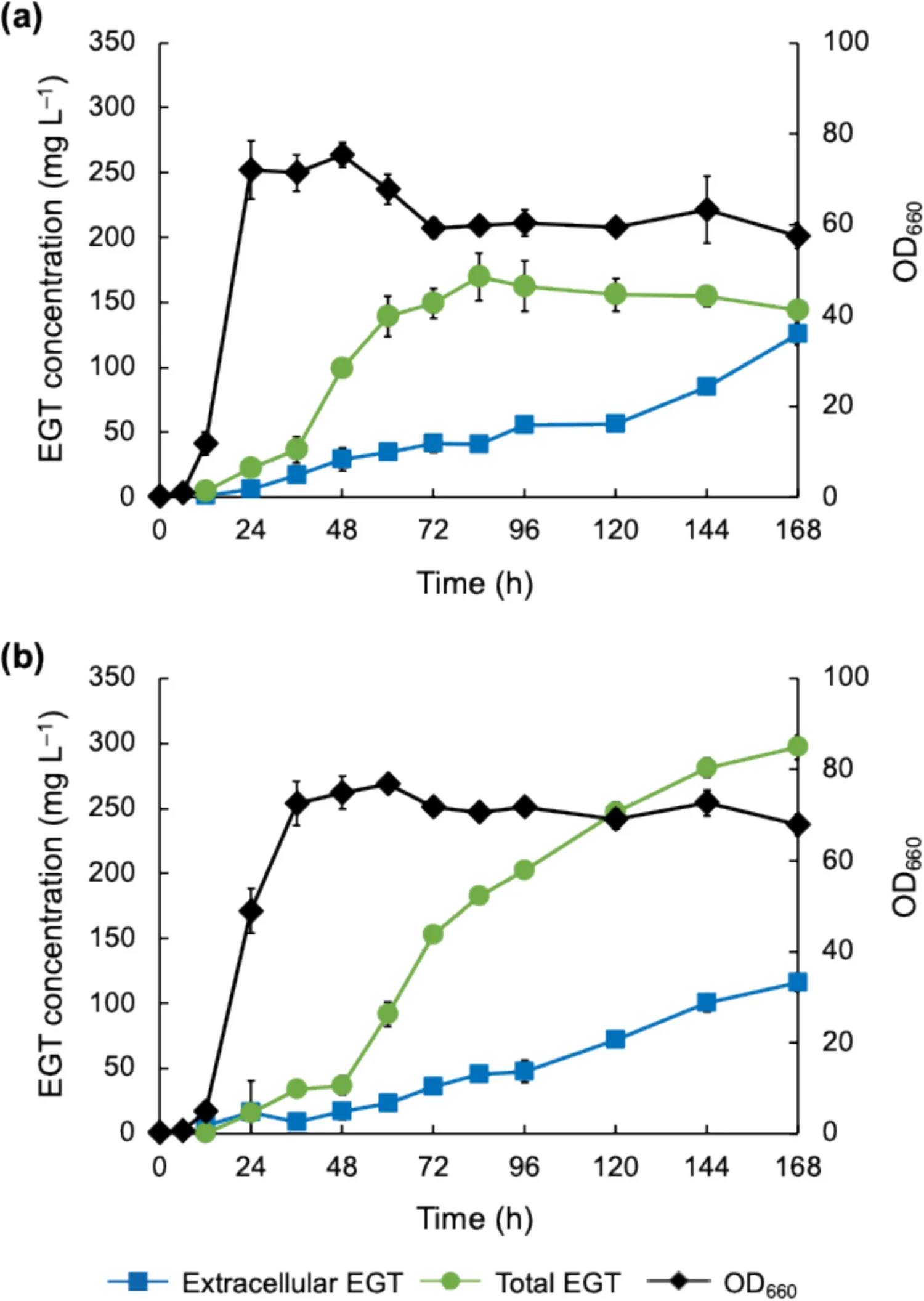
EGT production by the C strain under high osmotic pressure condition. The strain was cultured without (**a**) and with (**b**) 0.5 M sorbitol addition. Time courses of extracellular EGT concentration (squares), total EGT concentration (circles), and OD_660_ (diamonds) are shown. The experiment was conducted in triplicate. Average ± standard deviation is shown

Next, we investigated the timing of the induction of EGT accumulation as the osmotic pressure increased. Figure 4 shows the time course of cell growth and the intracellular and extracellular concentrations of EGT in the C strain with and without 0.5 M sorbitol addition. Extracellular EGT concentration at the end of cultivation (168 h) was not changed by 0.5 M sorbitol addition (*p* = 1.8 × 10^−1^ in *t*-test), suggesting that the EGT secretion may not be affected by osmotic pressure. Interestingly, with 0.5 M sorbitol addition, the total EGT production continued to increase until the end of culture, and it was significantly higher than that without sorbitol addition at the end of cultivation (*p* = 2.1 × 10^−5^ in *t*-test). Total EGT production rate in the presence of 0.5 M sorbitol, notably, decreased after 72 h, but total EGT production continued until 168 h. On the other hand, without sorbitol addition, total EGT production stopped between 72 and 84 h.

To investigate whether increased EGT production in the C strain under high osmotic pressure conditions was caused by the enhancement of transcription of EGT biosynthetic genes from the *tuf* promoter on the pECt-Mp_egtB-Ms_egtDE plasmid, we conducted reverse transcription-quantitative PCR (RT-qPCR) analysis of the Mp_*egtB* and Ms_*egtE* genes in the C strain cultured with 0.5 M sorbitol. The relative expression levels of EGT synthetic genes in the C strain with 0.5 M sorbitol addition were slightly higher than those without sorbitol addition, but the increased levels were small (Supplementary Fig. S6), suggesting that increased EGT production by sorbitol addition is not caused by enhancement of transcription of EGT biosynthesis genes.

### Effect of osmotic pressure on EGT production by CHS2 strain in batch and fed-batch cultivations

**Fig. 5:**
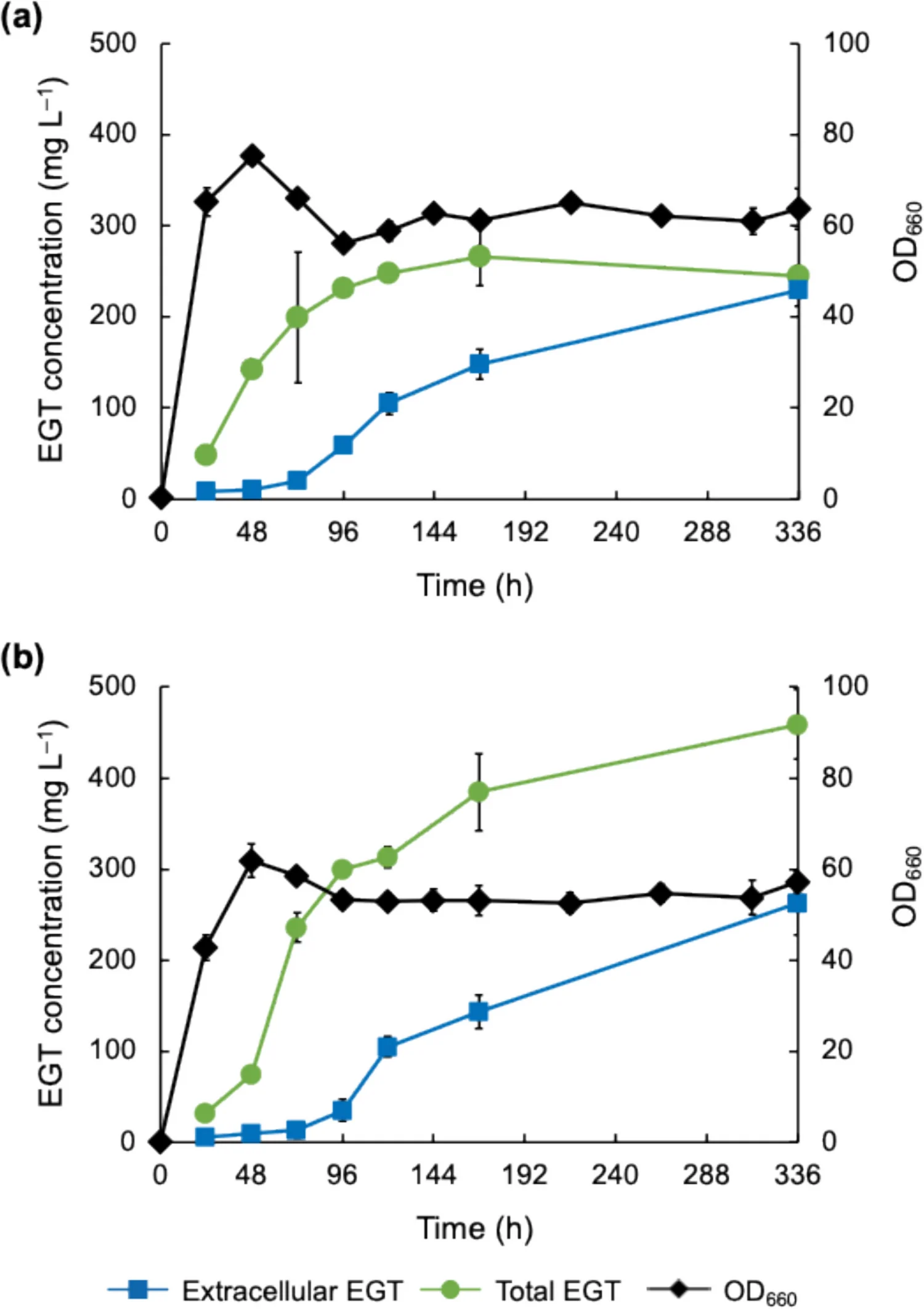
EGT production by the CHS2 strain under the osmotic pressure condition. The strain was cultured without (**a**) and with (**b**) 0.5 M sorbitol addition. Time courses of extracellular EGT concentration (squares), total EGT concentration (circles), and OD_660_ (diamonds) are shown. The experiment was conducted in triplicate. Average ± standard deviation is shown

The recombinant strain CHS2, in which the biosynthesis of cysteine, histidine, and SAM was modulated, was evaluated for EGT productivity under high osmotic pressure conditions in batch cultivation for 336 h (Fig. 5). The extracellular EGT concentration for CHS2 with 0.5 M sorbitol addition was similar to that without sorbitol addition in the first 168 h. Subsequently, it was slightly higher than that without sorbitol addition at 336 h, but this increase was not significant (*p* = 2.1 × 10^−1^ in *t*-test). This result indicates that EGT secretion in the CHS2 strain as well as the C strain is not significantly affected by the increase in osmotic pressure. Total EGT production (i.e., intracellular plus extracellular EGT amounts per culture volume) in the CHS2 with 0.5 M sorbitol addition reached 385 and 459 mg L^–1^ at 168 and 336 h, respectively, which was higher than that in the previous research on EGT production using *C. glutamicum* as a host (Kim et al. 2022). In contrast, without sorbitol addition, the increase in total EGT production started declining at 72 h and stopped after 120 h. The total EGT concentration with 0.5 M sorbitol addition at 336 h was significantly higher than that without sorbitol addition (*p* = 7.6 × 10^−4^ in *t*-test).

**Fig. 6:**
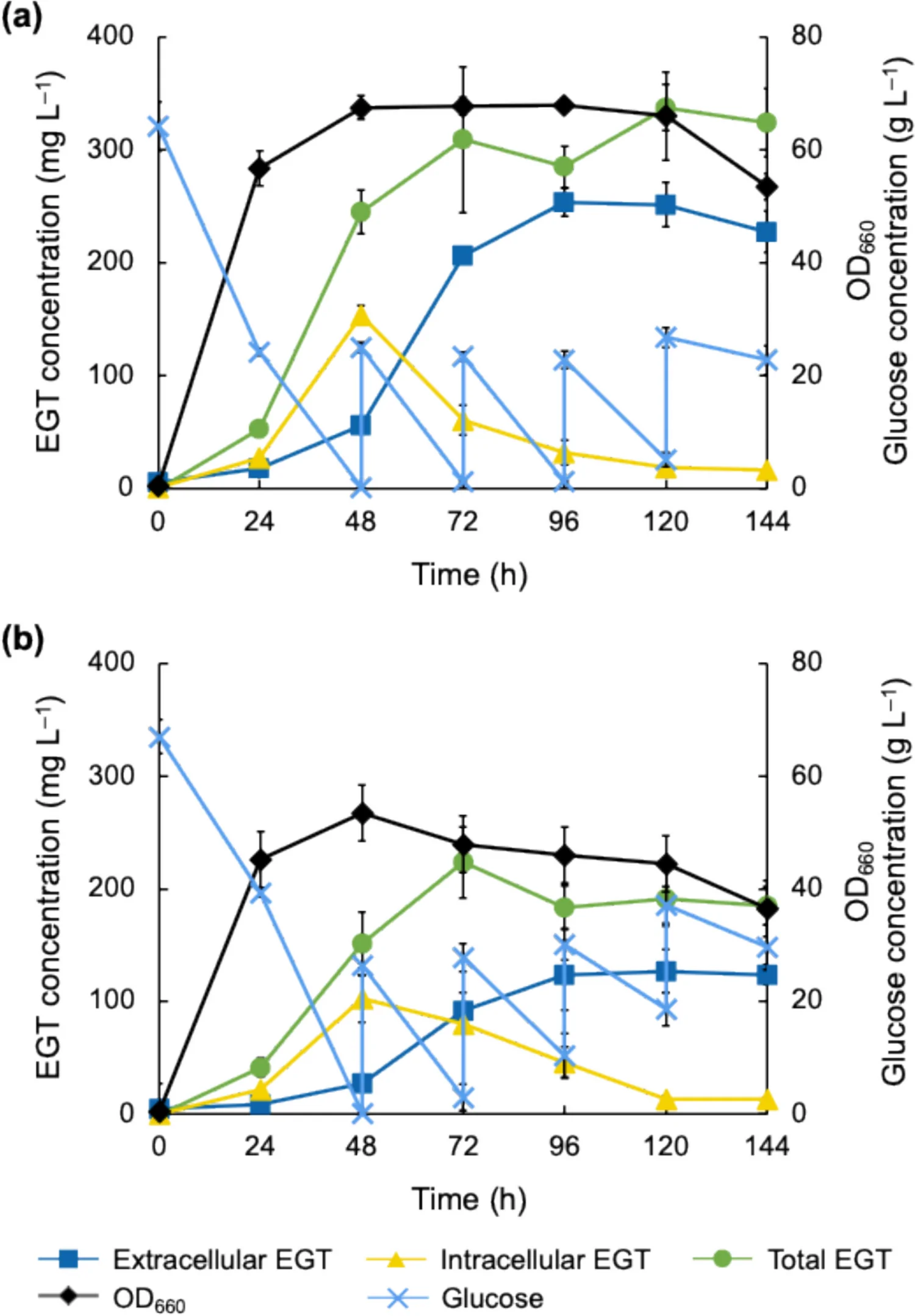
EGT production in fed-batch culture of the CHS2 strain. The CHS2 was cultured without (**a**) and with (**b**) 0.5 M sorbitol addition. Time courses of extracellular EGT concentration (squares), intracellular EGT concentration (triangles), total EGT concentration (circles), OD_660_ (diamonds), and glucose concentration in culture supernatant (crosses) are shown. The experiment was conducted in triplicate. Average ± standard deviation is shown

Furthermore, we conducted fed-batch cultivation of the CHS2 strain (Fig. 6). In the medium without sorbitol addition, the extracellular concentration and total EGT production reached 253 and 285 mg L^–1^, respectively, at 96 h, indicating that the superior EGT productivity and EGT secretion were achieved in the fed-batch cultivation more rapidly than in batch cultivation. Upon the addition of 0.5 M sorbitol, glucose consumption was delayed and cell growth was reduced compared to those without sorbitol addition. Moreover, the addition of sorbitol significantly reduced EGT production by the CHS2 strain during fed-batch cultivation (*p* = 2.7 × 10^–3^ and 2.5 × 10^–3^ for total and extracellular EGT concentration at 96 h, respectively, in *t*-test).

### EGT production by the strains lacking betaine transporters

*C. glutamicum* possesses betaine and proline importers, which are activated under high osmotic stress. According to Steger et al. (2004), these importers are activated by both NaCl and sorbitol. They also reported that the importers exhibit maximal transport activity in the presence of 1 M sorbitol. It was expected that preventing the incorporation of EGT into cells (probably via BCCT) would improve EGT productivity in *C. glutamicum* harboring heterologous EGT biosynthesis pathways. Therefore, we investigated the effect of deletion of the *betP*, *lcoP*, *ectP*, and *proP* genes encoding compatible solute importers, which were activated by increasing osmotic pressure, on EGT production in the C strain with 0.5 M sorbitol addition.

We initially examined the effect of *betP* gene deletion on EGT production in the C strain with 0.5 M sorbitol addition because BetP is the most active betaine transporter in *C. glutamicum*. The extracellular, intracellular, and total EGT concentrations were measured at 168 and 336 h. As shown in the Supplementary Fig. S6, *betP* deletion slightly increased the extracellular EGT concentration compared to that in the C strain; this increase was significant (1.5 × 10^−2^ and 1.7 × 10^−2^ for the extracellular EGT concentration at 168 and 336 h, respectively, in *t*-test). The intracellular EGT concentration in the CΔ*betP* strain was a bit higher than that in the C strain at 168 h and 336 h (*p* = 1.1 × 10^−2^ and 3.7 × 10^−2^, respectively, in *t*-test), but their difference became small at 336 h. These results indicated that the deletion of *betP* affected EGT accumulation, and EGT productivity was marginally improved by the deletion of *betP*.

Furthermore, we examined the effect of deletion of *lcoP*, *ectP*, and *proP* genes encoding other betaine transporters on EGT production in the C strain with 0.5 M sorbitol addition (Supplementary Fig. S6). One-way ANOVA of EGT production data revealed that the deletion of these genes did not affect EGT production (*p* = 7.2 × 10^−2^, 7.0 × 10^−2^, 7.3 × 10^−2^, and 8.2 × 10^−1^ for extracellular EGT concentration at 168 and 336 h and intracellular EGT concentration at 168 and 336 h, respectively). However, a trend of increased extracellular EGT concentration by deletion of *lcoP* and *ectP* was observed at 336 h. Although the intracellular EGT concentration in CΔ*lcoP* strain at 168 h was higher than that in the C strain, it became similar at 336 h; a similar phenomenon was observed in the CΔ*betP* strain. These results suggest that the deletion of *lcoP* and *ectP* might affect EGT production in *C. glutamicum*.

## Discussion

In this study, through strain breeding strategies and the medium optimization, we achieved the highest EGT production (459 mg L^−1^; intracellular plus extracellular EGT concentration) among previous studies using *C. glutamicum* as a host. Based on our findings on EGT production using *C. glutamicum*, increasing the osmolarity of the culture medium as well as modulating metabolism related to substrate supply for EGT biosynthesis may enhance EGT productivity in fermentation processes using other microorganisms.

The EGT concentration in the culture supernatant increased by approximately twofold through modulation of histidine and SAM biosynthesis using metabolic engineering (Fig. 2). Additionally, total EGT production (i.e., the summation of intracellular and extracellular EGT amounts per culture volume) increased by 1.7-fold (Fig. 5). However, in the present study, the insertion of the *tuf* promoter just upstream of the *hisEG* operon negatively affected cell growth and EGT production of *C. glutamicum*. This result may be caused by excess histidine formation due to the overexpression of *hisEG*, suggesting that histidine overaccumulation may be toxic to cells. Notably, cell growth reduction was not observed in the HS1 and CHS1 strains, in which *the metK* gene was overexpressed to enhance SAM supply together with *hisEG* overexpression, whereas it was significant in the H1 and CH1 strains (Supplementary Fig. S3), suggesting that histidine toxicity might be relieved by the conversion of histidine to hercynine.

As shown in Fig. 3, the time courses of the intracellular and extracellular EGT concentrations revealed that the EGT produced during approximately 84 h primarily accumulated in the C strain, assuming that intracellular EGT was slowly secreted from cells for about 2 weeks. According to the report by van der Hoek et al. (2019) on EGT production by *S. cerevisiae*, EGT leakage from *S. cerevisiae* cells was likely due to cell death because the reduction in culture turbidity coincided with an increase in extracellular EGT concentration. However, in our study, the extracellular EGT concentration was independent of changes in OD_660_. This phenomenon suggests that the secretion of EGT from *C. glutamicum* cells is likely mediated by native transporter(s) that are specific for other substrates. Although active EGT secretion and the existence of EGT-specific exporter(s) in *M. smegmatis* were suggested (Emani et al. 2013), no such transporters have been identified in any organism. The identification of EGT-specific exporters is crucial for efficient EGT production.

Compared to previous studies on bioproduction using *C. glutamicum*, the cultivation period in this study (168–336 h) is relatively long. As observed in some previous studies on bioproduction using *C. glutamicum* (Lai et al. 2012; Litsanov et al. 2012; Wieschalka et al. 2012), most of the recombinant *C. glutamicum* strains constructed in this study exhibited a decline in OD_660_ from 48 to 72 h. However, after 72 h, OD_660_ values remained almost constant to the end of the cultivation period and the EGT secretion continued until the end of cultivation (Figs. 3a, 4, 5, 6, and S3). As for EGT production, the time courses of total EGT production (Figs. 4a and 5a), which correspond to the levels of EGT biosynthesis, showed a steady increase until 72 h and then remained constant. On the other hand, extracellular EGT concentration (Figs. 4a and 5a), which corresponds to EGT secretion, continued increasing gradually even after the cell growth stopped at 72 h. The results suggest the recombinant strains can secrete EGT that is synthesized until 72 h, despite the absence of further cell growth and EGT biosynthesis. We speculate that the prolonged cultivation may be supported by the cell robustness. *C. glutamicum* is known for its robustness against environmental perturbations that can affect the productivity of the target compounds during bioproduction processes (Liu et al. 2021). It is thought that such robustness of *C. glutamicum* is related to the existence of a mycolic acid-containing cell surface layer outside the peptidoglycan cell wall (Laneelle et al. 2013). Moreover, in the present study, EGT itself as well as mycothiol, which is a native antioxidant in *C. glutamicum* (Liu et al. 2013; Hartmann et al. 2020), may have contributed to cellular stability by mitigating oxidative stress during the later stages of cultivation. In addition, CaCO_3_ added to the medium may have helped maintain an extracellular pH stably. These factors may have supported the prolonged cultivation of the EGT-producing recombinant strains of *C. glutamicum*.

For most bacteria, intracellular accumulation of betaine as a compatible solute is an effective strategy against high osmotic stress. EGT has a betaine structure and is not toxic to cells like other betaines, suggesting a potential role in protecting cells against high osmotic stress by reducing turgor pressure and stabilizing enzymes (Arakawa and Timasheff 1985). In the present study, EGT production by the C strain was enhanced approximately twofold by culturing the cells under high osmotic pressure conditions (Figs. 3 and 4). The same phenomenon was observed in the CHS2 strain (Fig. 5). Additionally, EGT production by the C strain continued for 72 h without sorbitol addition (Fig. 4a), whereas the period of EGT production in CHS2 was extended to 120 h (Fig. 5a), suggesting that metabolic engineering related to histidine and SAM supply increases EGT production, and the period of EGT production is extended. Cultivation under high osmotic pressure extended the period of EGT production in both C and CHS2 strains; EGT production in both strains continued for 168 h, which was longer than that without sorbitol addition (Figs. 4b and 5b). Since RT-qPCR analysis revealed that the transcription of genes related to heterologous EGT biosynthesis in *C. glutamicum* cells was not activated under high osmotic pressure conditions (Supplementary Fig. S6), the mechanism for increasing EGT production by *C. glutamicum* under high osmotic pressure conditions is obscure. However, it is known that *C. glutamicum* exhibits increased biosynthesis of trehalose and proline, activation of glycolysis and the TCA cycle, and enhanced ATP production as inherent responses to osmotic stress, but the pentose phosphate pathway is not affected by osmotic stress (Varela et al. 2003). Activation of the glycolysis and TCA cycle may enhance biosynthesis of methionine and cysteine, both of which are necessary for EGT biosynthesis. Enhanced ATP supply may contribute to the enhancement of sulfate reduction leading to biosynthesis of sulfur-containing amino acids (i.e., methionine and cysteine), and biosynthesis of phosphoribosyl pyrophosphate required for histidine and SAM biosynthesis. The increase in osmotic pressure would lead to enhanced supply of compounds that are necessary for EGT biosynthesis, such as methionine, cysteine, histidine, and SAM. Hence, increased EGT production under high osmotic pressure conditions might be attributed to such metabolic changes in *C. glutamicum*.

According to the time course data of EGT production by the C and CHS2 strains (Figs. 4 and 5), during the first 72 h, total EGT production with sorbitol addition was comparable to that without sorbitol addition in both strains. After 72 h, however, total EGT production with sorbitol addition was higher than that without sorbitol addition in both strains until the end of cultivation, indicating that EGT production is enhanced by increasing osmotic pressure. On the other hand, EGT secretion was not significantly affected by 0.5 M sorbitol addition in the C and CHS2 strains (Figs. 4 and 5). Particularly, as shown in Fig. 5b, the cells could continue secreting EGT up to 336 h without reaching a plateau, as long as the intracellular pool of EGT remained high enough. These results indicate that high osmotic pressure does not affect EGT secretion. Considering the increased total EGT production and unaffected EGT secretion by sorbitol addition, it is thought that the additionally produced EGT under high osmotic pressure conditions would be reflected in intracellular EGT levels, whereas extracellular EGT levels remained unchanged. It is speculated that *C. glutamicum* does not possess an EGT-specific transporter and EGT secretion is unregulated because EGT is not a native metabolite for *C. glutamicum*. Moreover, since EGT is an amino acid with a betaine structure, it is also unlikely to pass through the membrane by diffusion. Therefore, it is thought that EGT secretion may not be regulated to maintain the intracellular levels of EGT and that EGT may be exported through membrane channel(s).

According to Steger et al. (2004), betaine and proline importers can be activated by sorbitol in *C. glutamicum*, and these importers exhibit maximal transport activity in the presence of 1 M sorbitol. Recently, OCTN1 and EgtU from human and Firmicutes, respectively, have been identified as transporters for EGT uptake (Gründemann et al. 2005; Zhang et al. 2022). Interestingly, both transporters were initially identified as importers of quaternary amines. Given the structural similarity between EGT and betaines (Fig. 1), EGT may be incorporated via four osmotic stress-responsive transporters in *C. glutamicum* such as BetP, LcoP, EctP, and ProP. To investigate the impact of these transporters on EGT productivity, we investigated the effect of deletion of the genes encoding the transporters in the C strain on EGT production (Supplementary Fig. S7). These deletions did not affect cell growth (data not shown), but the deletion of *betP* marginally increased EGT production. These results indicate that BetP may be responsible for EGT import in *C. glutamicum*. In addition, a trend of slightly increased EGT production by deleting *lcoP* and *ectP* genes in the C strain was observed as well (Supplementary Fig. S7). The loss of function of an importer may be compensated by the function of other BCCTs. Alternatively, these transporters may exhibit low affinity for EGT. ProP, which does not belong to the BCCTs family, is a member of the H^+^ symporter family. Peter et al. (1998) previously showed lower expression and betaine import activity of ProP opposed to BCCTs. Thus, it is suggested that ProP does not exhibit EGT transport activity.

In fed-batch cultivation of the CHS2 strain without sorbitol addition, glucose consumption continued for 120 h and the OD_660_ remained stable. While the total EGT production was approximately 250 mg L^−1^ in both batch and fed-batch cultures, the secretion rate was faster in fed-batch cultivation than in batch cultivation; the extracellular EGT concentration reached 206 mg L^−1^ at 72 h in fed-batch cultivation, whereas it took 336 h to secrete approximately 200 mg L^−1^ of EGT in batch cultivation. As discussed above, EGT secretion from *C. glutamicum* cells appears to be unregulated during batch culture. However, the repeated increase and decrease in osmotic pressure caused by the addition of glucose and ammonium sulfate and their consumption by cells may actively promote EGT secretion. Such nutrient supply and consumption may cause the activation of mechanosensitive channels, which are responsible for the export of compatible solutes in response to osmotic downshift (Booth 2014). Thus, as the supplied nutrients are consumed, the osmotic pressure decreases, and consequently, EGT secretion might be promoted via mechanosensitive channels. In contrast, in fed-batch cultivation of the CHS2 strain under high osmotic pressure conditions created by sorbitol addition, glucose consumption declined, and the OD_660_ gradually decreased after 72 h. The CHS2 strain may not tolerate the total osmolarity of the culture broth containing sorbitol. The addition of glucose and ammonium sulfate leads to an increase in osmolarity corresponding to approximately 0.22 and 0.34 osmol L^−1^, respectively. Such an increase in osmotic pressure may damage the cells, and as a result, EGT production may decrease in fed-batch cultivation with 0.5 M sorbitol addition.

In this study, we improved EGT production in *C. glutamicum* by expressing heterologous EGT biosynthesis genes using engineered histidine and SAM biosynthesis and established an EGT production system achieving the highest EGT production levels among the studies in *C. glutamicum*. Notably, we found that an increase in osmotic pressure effectively improved the EGT titer. The recombinant EGT-producing strains of *C. glutamicum* used in this study may actively produce and accumulate EGT in cells as a compatible solute. However, the growth property of the EGT-producing strain was similar to that of the empty vector-carrying CYS-2 strain under high osmotic pressure conditions created by NaCl and sorbitol addition (Supplementary Fig. S5), suggesting EGT may not function as a compatible solute. Further analyses are required to understand the mechanisms underlying the enhancement of EGT production under high osmotic pressure conditions. It is important to evaluate whether EGT functions as a non-native compatible solute; the effect of externally added EGT on cell growth under osmotic stress should be investigated as previously described by (Pérez-García et al. 2019).

Additionally, an increase in osmotic pressure in the culture can reduce the risk of microbial contamination during fermentation. EGT production using high osmolarity media is expected to be more efficient, economical, and eco-friendly. EGT is widely distributed among various species, and its specific transporters have been identified. Our findings provide new insights into the biological roles of EGT, not only as an antioxidant but also as a compatible solute.

## Supplementary Information

Below is the link to the electronic supplementary material.

ESM 1 (PDF 413 KB)

## Data Availability

All data generated or analyzed in this study are included in the published article and its supplementary information file.
