# Supplementary material for: Enhancement of ergothioneine production in Corynebacterium glutamicum by increasing osmotic pressure
Source: Appl Microbiol Biotechnol. 2025 Dec 8;109(1):255. doi: 10.1007/s00253-025-13639-3 (PMC12689799; doi:10.1007/s00253-025-13639-3)
Supplement: Supplementary file 1 — (PDF 413 KB) [file 253_2025_13639_MOESM1_ESM.pdf]

Supplementary materials

Enhancement of ergothioneine production in *Corynebacterium glutamicum* by increasing osmotic pressure

Yuno Takahashi[†] · Takashi Hirasawa[*]

School of Life Science and Technology, Institute of Science Tokyo, 4259 Nagatsuta-cho, Midori-ku, Yokohama, Kanagawa 226-8501, Japan

[†]Current address: Department of Natural Environmental Studies, Graduate School of Frontier Sciences, The University of Tokyo, 5-1-5 Kashiwanoha, Kashiwa, Chiba 277-8564, Japan

[*]Corresponding author

Takashi Hirasawa

thirasawa@life.isct.ac.jp

ORCID

Takashi Hirasawa: 0000-0002-5183-2953

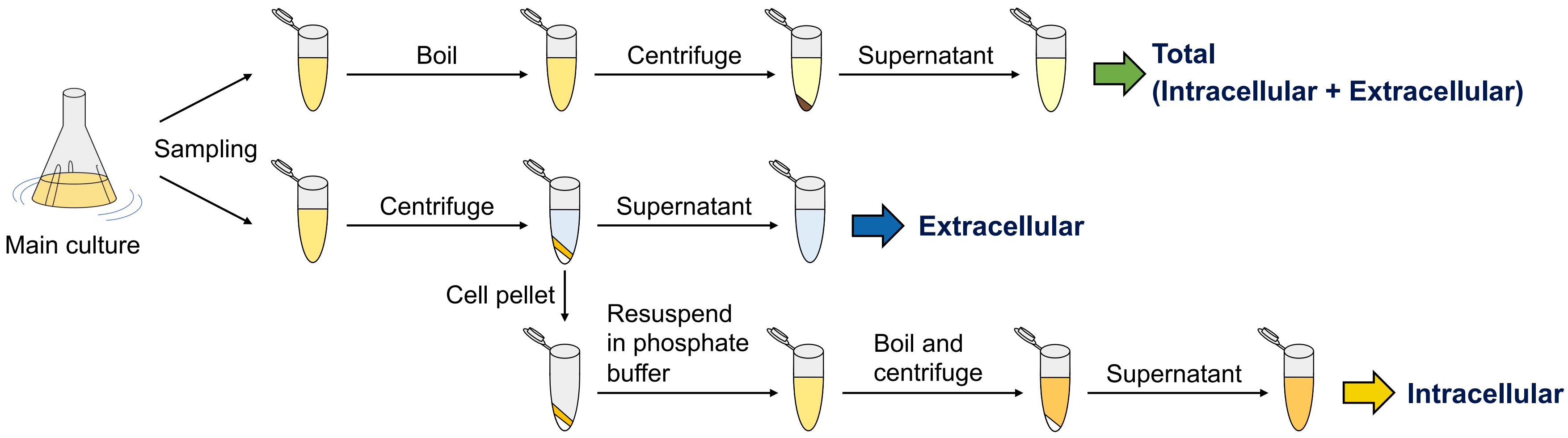

Supplementary Fig. S1 Procedures to collect samples from culture of the EGT-producing strains of *C. glutamicum*.

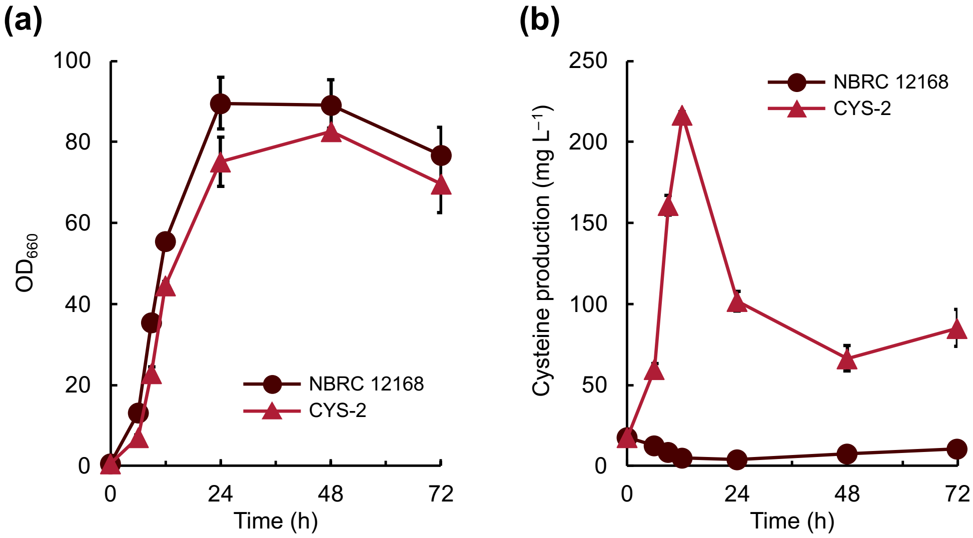

Supplementary Fig. S2 Cysteine production by *C. glutamicum* CYS-2 strain. The wild-type strain NBRC 12168 and cysteine-producing strain CYS-2 were cultured in a semisynthetic medium. Time courses of cell growth (a) and cysteine production (b) of the NBRC 12168 (circles) and CYS-2 (triangles) are shown. Average ± standard deviation in triplicate experiments is shown.

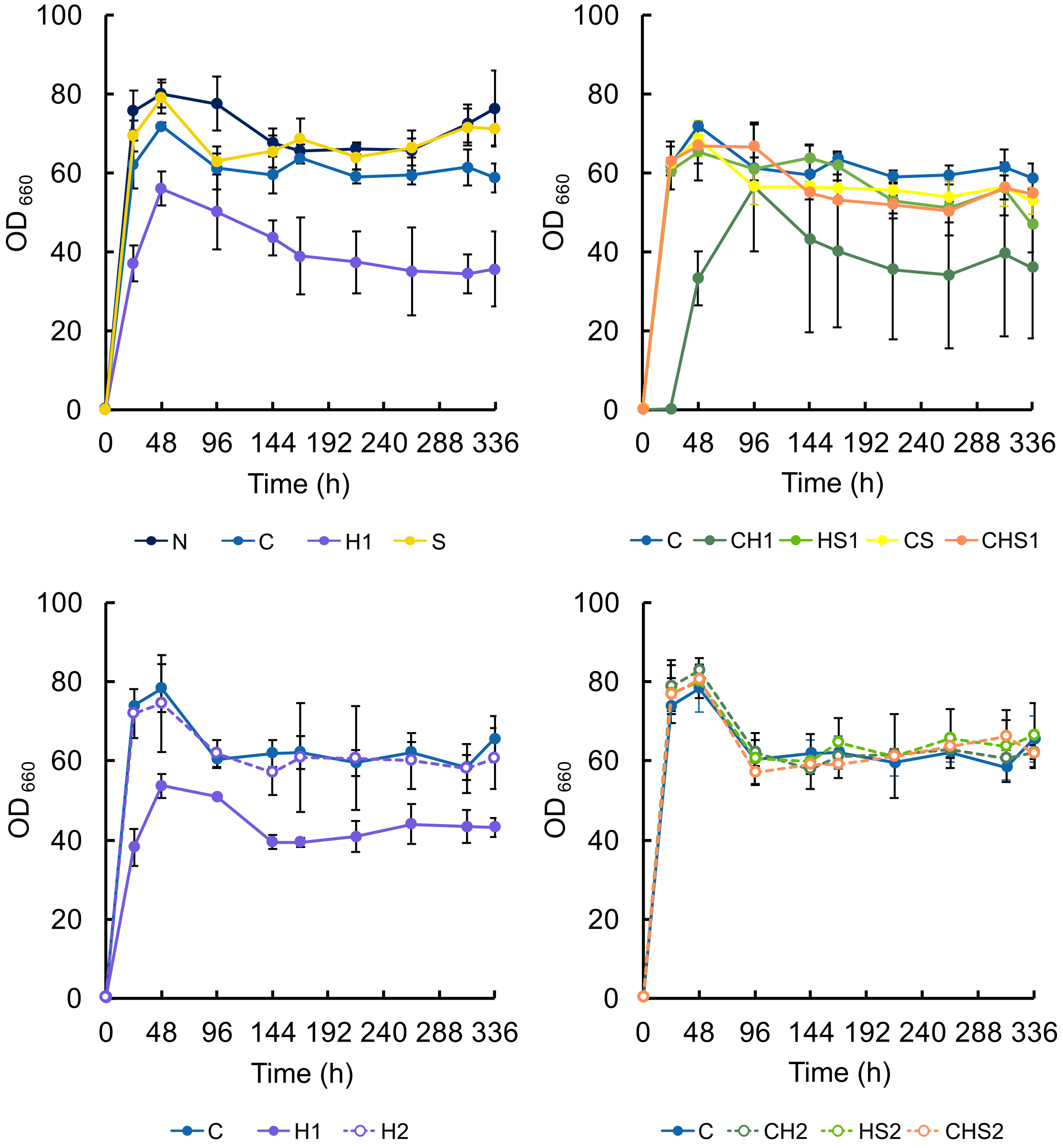

Supplementary Fig. S3 Time courses of cell growth of *C. glutamicum* where biosynthesis of cysteine, histidine and SAM was engineered. The strains harboring pECt-Mp_egtB-Ms_egtDE were cultured in the semisynthetic medium for 336 h. Average ± standard deviation in triplicate experiments is shown.

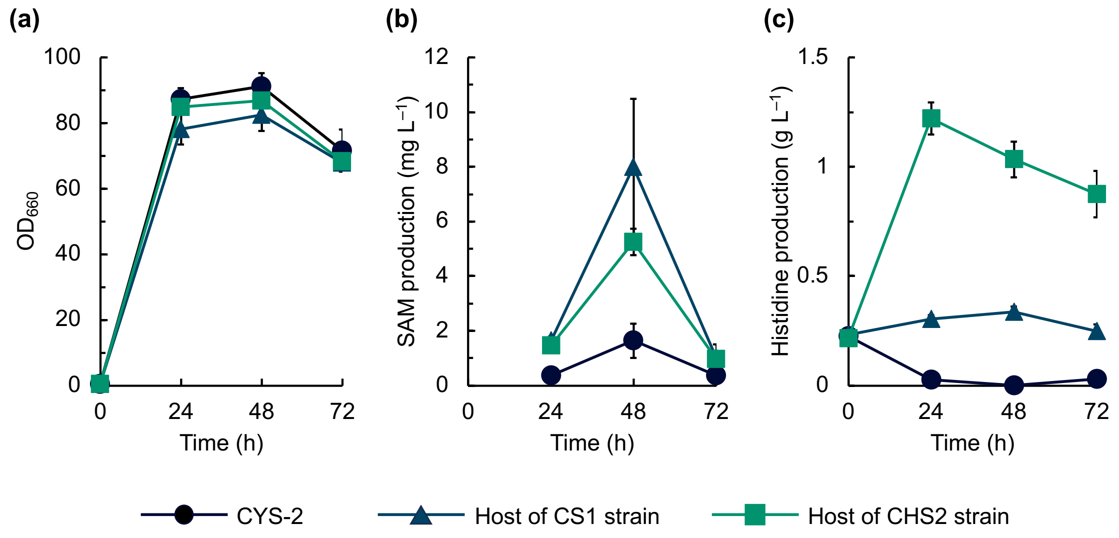

Supplementary Fig. S4 Effect of engineering of *S*-adenosylmethionine and histidine production in *C. glutamicum*. The CYS-2, host of CS1 strain and host of CHS2 strain, all of which are not transformed with the pECt-Mp_egtB-Ms_egtDE plasmid, were cultured in a semisynthetic medium. Time courses of cell growth (a), *S*-adenosylmethionine (SAM) production (b) and histidine production (c) of the CYS-2 (circles), host of CS1 strain (triangles) and host of CHS2 strain (squares) are shown. Average ± standard deviation in triplicate experiments is shown.

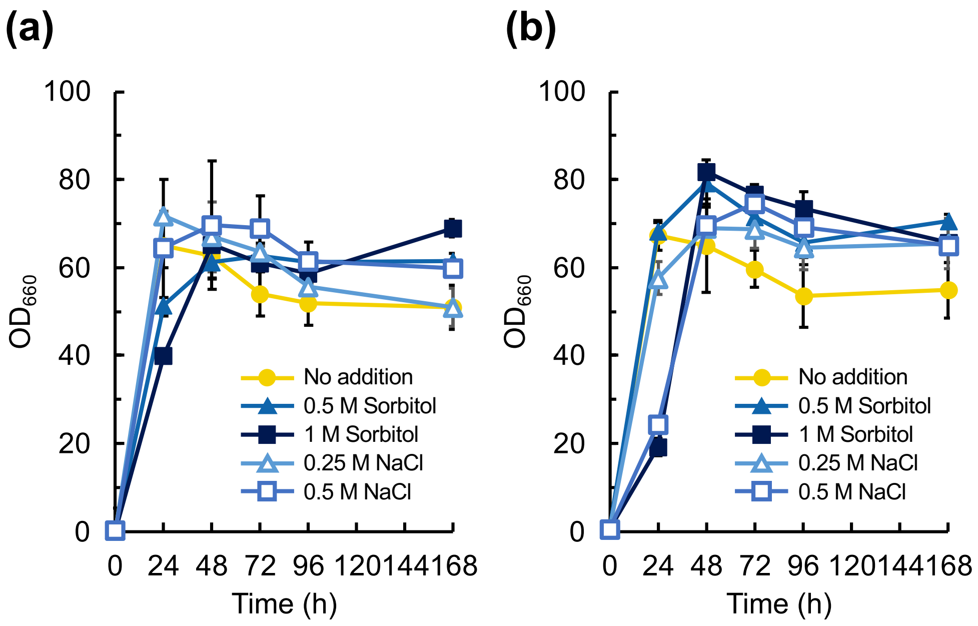

Supplementary Fig. S5 Effect of high osmotic pressure on the growth of the C strain. The CYS-2/pECt and C strains were cultured under high osmotic pressure conditions Cell growth of the CYS-2/pECt (a) and C (b) strains without addition of sorbitol and NaCl (filled circles) and with addition of 0.5 (filled triangles) and 1.0 (filled squares) M sorbitol and 0.25 (open triangles) and 0.5 M (open squares) is shown. Average ± standard deviation in triplicate experiments is shown.

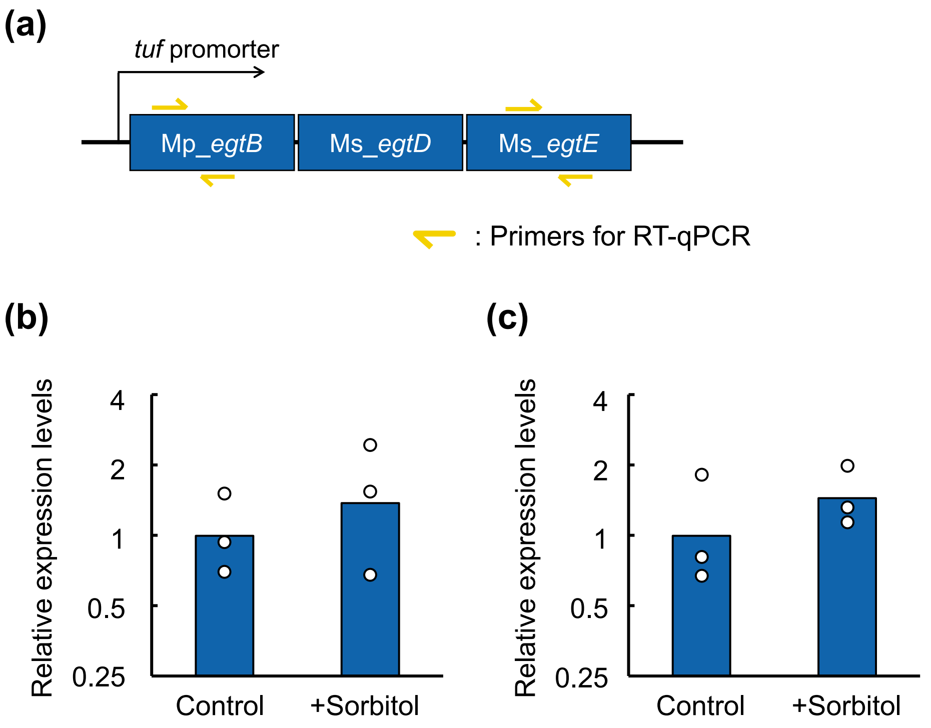

Supplementary Fig. S6 Effect of high osmotic pressure on expression of heterologous EGT biosynthetic genes in *C. glutamicum*. The C strain was cultured with or without addition of 0.5 M sorbitol. RNA samples were extracted from the cells collected at 12 h after starting cultivation. The primers which anneal to the target Mp_*egtB* and Ms_*egtE* genes on the pECt-Mp_egtB-Ms_egtDE were shown in (a). In addition, the geometric mean of fold changes (filled bars) and their individual values (open circles) in the expression of Mp_*egtB* and Ms_*egtE* in the C strain cultured with 0.5 M sorbitol (+Sorbitol) relative to those without sorbitol addition (Control) are shown in (b) and (c), respectively. The experiment was conducted in triplicate.

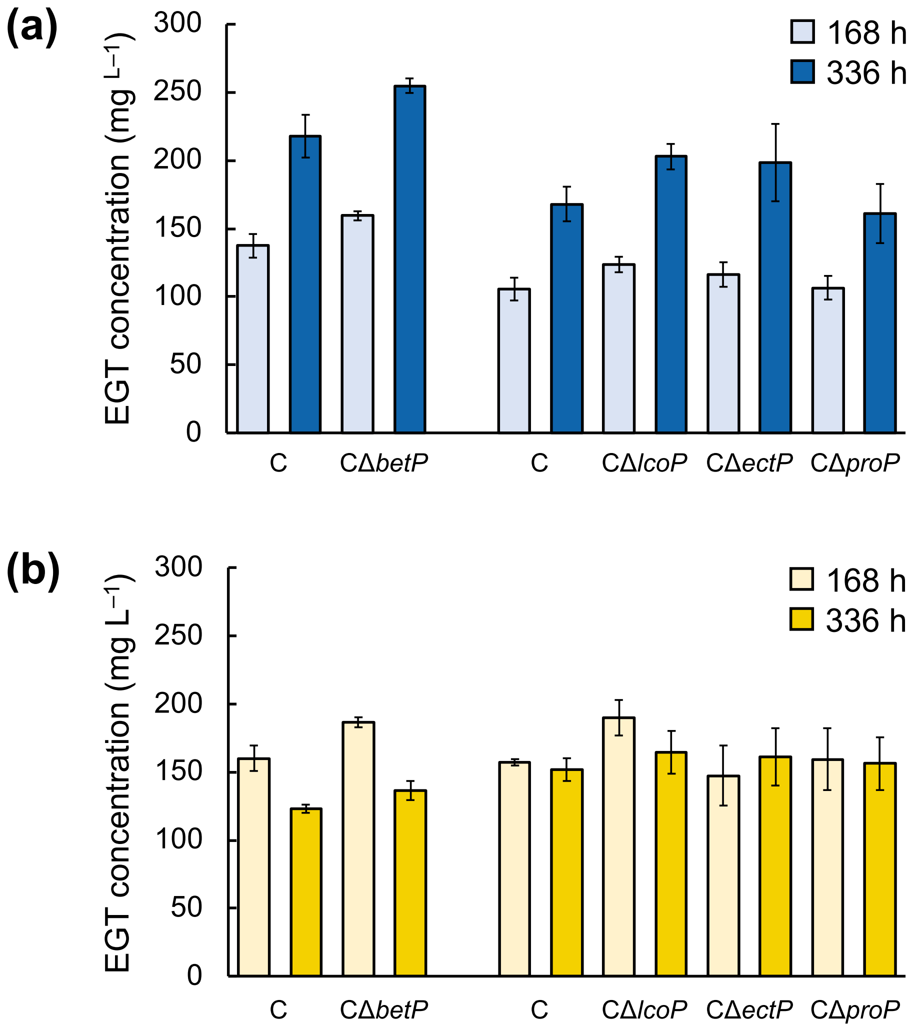

Supplementary Fig. S7 Effect of deletion of the genes encoding betaine transporters on EGT production in the C strain. The C strain and its deletion strains for betaine transporters, CΔ*betP,* CΔ*lcoP,* CΔ*ectP,* and CΔ*proP,* were cultured in the semisynthetic medium containing 0.5 M sorbitol for 336 h. Extracellular (a) and intracellular (b) concentrations of EGT at 168 and 336 h are shown. Average ± standard deviation in triplicate experiments is shown.

Supplementary Table S1 PCR primers for amplifying DNA fragments to construct plasmids which are used for modifying histidine and SAM biosynthesis

Target	Primer (5´→3´)	Restriction site for cloning into pK18*mobsacB*
Upstream of codon GCC for 270th Ala replacing with GAT for Asp in *hisG*	TTCCGAATTCTTCGGTTCCTCCACTTTCCG	EcoRI
	AAGCCAGGAT**ATC**TTCAGCGCCGAGTCCAGCAAG[a]	–
Downstream of codon GCC for 270th Ala replacing with GAT for Asp in *hisG*	GGCGCTGAA**GAT**ATCCTGGCTTCTGAAATCCG[a]	–
	CGAGGATCCACTGATCCATACAGCGTTCC	BamHI
Upstream of initiation codon of *hisE*	TTGGGATCCGCGGACTACGAGCGCTACC	BamHI
	CATTCGCAGGGTAACGGCCATCGGTACATTCTTCCACACAAC[b]	–
Downstream of initiation codon of *hisE*	GTCCAGGAGGACATACAATGAAGACATTTGACTCGCTG[b]	–
	CATAAGCTTGTGCTGCGTAACGGAAAGTG	HindIII
Upstream plus downstream of initiation codon of *metK*	GAGAGAATTCCGGTGGTGTCCACACAAGAC	EcoRI
	CCAGGATCCGACGTGTTCGCGCAGTTGGG	BamHI
pK18*mobsacB_metK* by inverse PCR	GCAGGGTAACGGCCAAAATACCCTTCTTTCGAAGAAGTTG[b]	–
	CCAGGAGGACATACAATGGCTCAGCCAACCGCCGTCC[b]	–
tuf promoter	TGGCCGTTACCCTGCGAATGTCCAC	–
	CATTGTATGTCCTCCTGGACTTCGTG	–

[a]Bold represents the codon GAT for Asp.

[b]Sequence with gray background represents the overlap region with the *tuf* promoter fragment.

Supplementary Table S2 PCR primers for amplifying upstream and downstream regions of the BCCT genes to construct the gene disruption plasmids

Target gene	Amplifying region	Primer (5′→3′)	Restriction site for cloning into pK18*mobsacB*
betP (NCgl0856)	Upstream	AAAAGAATTCGGTATGTTTCTTCGAATCTCC	EcoRI
		TAAAAATTGCAAAACTCACCTTTACTTGG	–
	Downstream	GGTGAGTTTTGCAATTTTTAACCCCTGATAC	–
		CCGGGATCCCGAGCTGGTTTCCGCAGTTGTCG	BamHI
lcoP (NCgl2251)	Upstream	CGTCGAATTCCATCCGCTGAAACGCCTGAAC	EcoRI
		TTATTTTTAGTGTGTCCTCTCCATTGTGCACC	–
	Downstream	GAGGACACACTAAAAAATAACGACTGGCTG	–
		GTAGGATCCGATCTTCACGCTCGCTTCG	BamHI
ectP (NCgl2230)	Upstream	CGATGAATTCCAAAATTCGGATGATTGAAG	EcoRI
		GGCTTTACGGGAGTAAAACCTCTCGTCATATC	–
	Downstream	GGTTTTACTCCCGTAAAGCCCGCTGCAAGGCG	–
		ATGCTGCAGGAACTATTACCTGACCACCAC	PstI
proP (NCgl2961)	Upstream	AAGGAGATATCCATACCCTTTATTTGGTGGCAG	EcoRV[a]
		AAGCAATCGTCTCATTCTTCCAATCAGTGGATAAC	–
	Downstream	GAAGAATGAGACGATTGCTTTTCGACGCACC	–
		GCTCTGCAGCAAATTGTCGACATGCTTCAC	PstI[a]

[a]The resulting fragment was cloned into SmaI–PstI sites of the pK18*mobsacB*.

Supplementary Table S3 Primers used for RT-qPCR

Target gene	Primer (5′→3′)
Mp_*egtB*	GATCGGACTCTATCACGAGCAG
	AAACGCCATTCGGCATCGTAGAC
Ms_*egtD*	CGTACACCTCACCGCATTGGC
	CCCGACATTGCAGTCCAGATG
16S rRNA	CTTACCTGGGCTTGACATGG
	CACCATAATGTGCTGGCAAC

Supplementary methods

Evaluation of productivity of cysteine by engineered *C. glutamicum*

For preparing the preculture, a single colony of the wild-type strain NBRC 12168 and cysteine-producing strain CYS-2 (Hirasawa et al. 2023) was inoculated into 5 mL L medium, which consists of 10 g L^{-1} hipolypepton (Shiotani M. S. Co., Ltd., Hyogo, Japan), 5 g L^{-1} dried yeast extract D-3H (Shiotani M. S. Co., Ltd.), 5 g L^{-1} NaCl, 1 g L^{-1} glucose (pH 7.0), and cultured at 30 °C for 1 days. Subsequently, 1.6 mL of the preculture was transferred into 40 mL semisynthetic medium, which is the same as that used for EGT production and constitutes of 80 g L^{-1} glucose, 30 g L^{-1} $(NH_4)_2SO_4$, 3 g L^{-1} $Na_2HPO_4 \cdot 12H_2O$, 6 g L^{-1} KH_2PO_4, 2 g L^{-1} NaCl, 84 mg L^{-1} $CaCl_2$, 3.9 mg L^{-1} $FeCl_3$, 0.9 mg L^{-1} $ZnSO_4 \cdot 7H_2O$, 0.3 mg L^{-1} $CuCl_2 \cdot 2H_2O$, 5.56 mg L^{-1} $MnSO_4 \cdot 5H_2O$, 0.1 mg L^{-1} $(NH_4)_6Mo_7O_{24} \cdot 4H_2O$, 0.3 mg L^{-1} $Na_2B_4O_7 \cdot 10H_2O$, 0.4 g L^{-1} $16MgSO_4 \cdot 7H_2O$, 40 mg L^{-1} $FeSO_4 \cdot 7H_2O$, 0.5 mg L^{-1} thiamin hydrochloride, 0.1 g L^{-1} ethylenediamine-*N*, *N*, *N′*, *N′* -tetraacetic acid disodium salt dihydrate, 0.03 mg L^{-1} D-biotin, 10 g L^{-1} dried yeast extract, and 25 g L^{-1} $CaCO_3$ (pH 7.2), in a 300-mL baffled flask for the main culture, and then the culture was incubated at 30 °C with rotary shaking at 200 rpm.

During cultivation, cell growth was monitored by measuring optical density of culture at 660 nm (OD_{660}) with a spectrophotometer UV-1280 (Shimadzu Corporation, Kyoto, Japan). The culture was diluted with 0.2 N HCl to dissolve $CaCO_3$, which is contained in culture broth, before OD_{660} measurement. Moreover, culture supernatant was obtained by centrifuging culture broth to measure cysteine concentration in culture supernatant. Cysteine concentration was measured based on the methods reported by (Gaitonde 1967); the protocol is described in the report by Kondoh and Hirasawa (2019).

Evaluation of productivity of histidine and *S*-adenosylmethionine by engineered *C. glutamicum*

The hosts of EGT-producing strains, C, CS1 and CHS2, all of which do not carry a plasmid pECt-P_{tuf}-Mp_*egtB*-Ms_*egtDE*, were cultured by the same method as that for evaluation of cysteine productivity described above. Culture both (300 μL) was centrifuged at 360 × *g* for 2 min to remove $CaCO_3$ from the culture and the supernatant was transferred to a new microcentrifuge tube, followed by centrifugation at 20000 × *g* for 2 min to pellet the cells. Then, the supernatant was transferred to a new microcentrifuge tube and the supernatant and cell pellet were stored at –30 °C.

For evaluation of histidine productivity, histidine concentration in the supernatant obtained after removing both $CaCO_3$ and cells was determined using high performance liquid chromatography (HPLC) system, Nexera lite (Shimadzu Co., Ltd., Kyoto, Japan) equipped with a refractive index detector, RID-20A (Shimadzu Co., Ltd.) and a cation chromatography column, Shodex IC YS-50 (Resonac Corporation, Tokyo, Japan). The column temperature was set to 40 °C. As a mobile phase, 6 mM phosphoric acid was

used. The flow rate of the mobile phase was set to 1 mL min^{-1}.
S-Adenosylmethionine (SAM) productivity in the hosts for EGT-producing strains was evaluated by determining intracellular SAM content. SAM extraction was performed based on the methods reported by (Han et al. 2015). Briefly, SAM was extracted from the cell pellet by suspending cells in 1.5 M perchloric acid and incubating at 4 °C for 24 h, followed by centrifugation of the suspension at 4 °C for 5 min. The extract was filtered through a 0.20-μm syringe filter DISMIC 13HP (Toyo Roshi Kaisha, Ltd., Tokyo, Japan). SAM concentration in the filtrates was determined the HPLC system with a photodiode array (PDA) detector, SPD-M40 (Shimadzu Co., Ltd.) through a column TSKgel ODS-80T_M (4.6 × 250 mm, 5 μm) (Tosoh Corporation, Tokyo, Japan) (She et al. 1994; Shibata et al. 2021). The column temperature was set to 35 °C. The mobile phase was 18% methanol (pH 3) containing 40 mM ammonium dihydrogen phosphate and 8 mM 1-heptanesulfonic acid sodium salt. The flow rate of the mobile phase was set to 0.5 mL min^{-1}. SAM was detected at 254 nm on the PDA detector. In the present study, SAM concentration per culture volume in the recombinant strains was determined.

Expression analysis of EGT biosynthesis genes using reverse transcription-quantitative PCR

Cells of the C strain cultured in the presence and absence of 0.5 M sorbitol in the semisynthetic production medium for 12 h were harvested by centrifugation and stored at –80 °C until RNA extraction. Total RNA was extracted from frozen cells using NucleoSpin RNA (Macherey-Nagel GmbH & Co., Düren, Germany). In reverse transcription (RT) and quantitative PCR (qPCR), ReverTra Ace qPCR RT Master Mix with gDNA Remover (Toyobo Co., Ltd., Osaka, Japan) and Thunderbird Next SYBR qPCR Mix (Toyobo Co., Ltd.), respectively, were used. In addition, a Thermal Cycler Dice Real Time System III (Takara Bio, Inc., Shiga, Japan) with the primer sets listed in Supplementary Table S3 were used for qPCR. The expression levels of the Mp_*egtB* and Ms_*egtD* genes in the presence of sorbitol relative to those in the absence of sorbitol was determined by the ΔΔCt method, using the Ct values for the Mp_*egtB* and Ms_*egtD* genes as targets and the 16S rRNA gene as a house-keeping gene.

References

Gaitonde MK (1967) A spectrophotometric method for the direct determination of cysteine in the presence of other naturally occurring amino acids. Biochem J 104:627-633. doi:10.1042/bj1040627

Han G, Hu X, Wang X (2015) Co-production of *S*-adenosyl-L-methionine and L-isoleucine in *Corynebacterium glutamicum*. Enzyme Microb Technol 78:27-33. doi:10.1016/j.enzmictec.2015.06.003

Hirasawa T, Shimoyamada Y, Tachikawa Y, Satoh Y, Kawano Y, Dairi T, Ohtsu I (2023) Ergothioneine production by *Corynebacterium glutamicum* harboring heterologous biosynthesis pathways. J Biosci Bioeng 135:25-33. doi:10.1016/j.jbiosc.2022.10.002

Kondoh M, Hirasawa T (2019) L-Cysteine production by metabolically engineered *Corynebacterium glutamicum*. Appl Microbiol Biotechnol 103:2609-2619. doi:10.1007/s00253-019-09663-9

She QB, Nagao I, Hayakawa T, Tsuge H (1994) A simple HPLC method for the determination of *S*-adenosylmethionine and *S*-adenosylhomocysteine in rat tissues: the effect of vitamin B_6 deficiency on these concentrations in rat liver. Biochem Biophys Res Commun 205:1748-1754. doi:10.1006/bbrc.1994.2871

Shibata Y, Takahashi T, Morimoto T, Kanai M, Fujii T, Akao T, Goshima T, Yamada T (2021) Quantitative stability of the folates highly accumulated in a non-Kyokai sake yeast. J Gen Appl Microbiol 67:214-219. doi:10.2323/jgam.2021.03.002
